# An iterative approach to evaluating impact of CTSA projects using the translational science benefits model

**DOI:** 10.3389/frhs.2025.1535693

**Published:** 2025-05-20

**Authors:** Kera Swanson, Nicole A. Stadnick, Isaac Bouchard, Zeying Du, Lauren Brookman-Frazee, Gregory A. Aarons, Emily Treichler, Maryam Gholami, Borsika A. Rabin

**Affiliations:** ^1^ UC San Diego Altman Clinical and Translational Research Institute Dissemination and Implementation Science Center, University of California San Diego, La Jolla, CA, United States; ^2^ Department of Psychiatry, University of California San Diego, La Jolla, CA, United States; ^3^ Child and Adolescent Services Research Center, University of California San Diego, La Jolla, CA, United States; ^4^ Department of Psychology, California State University, Northridge, CA, United States; ^5^ Jennifer Moreno Department of Veterans Affairs Medical Center, San Diego, CA, United States; ^6^ UC San Diego Altman Clinical and Translational Research Institute, University of California San Diego, La Jolla, CA, United States; ^7^ Herbert Wertheim School of Public Health and Human Longevity Science, University of California San Diego, La Jolla, CA, United States

**Keywords:** clinical and translational science, dissemination, evaluation, translational science benefits model (TSBM), impact

## Abstract

**Introduction:**

Demonstrating the relevance and impact of translational research across diverse settings is crucial making the research-to-practice pipeline more efficient. The Translational Science Benefits Model (TSBM) is a framework used to report societal and health impacts of clinical and translational research.

**Methods:**

A four-phase process was used to co-develop 12 TSBM Impact Profiles aimed at evaluating the impact of clinical and translational research and disseminating this information among diverse audiences. Content analysis was used to understand common and unique themes related to the TSBM domains and benefits across 12 projects.

**Results:**

Across the 12 TSBM Impact Profiles, TSBM benefits covered all four TSBM domains (Clinical, Community, Economic, and Policy), with a notable focus on Clinical and Community-related benefits. TSBM Impact Profiles took an average of 9 h to complete, with each phase taking 1–3 h to complete. Common themes included Clinical Innovation and Care Integration, Advancing Health Equity and Accessibility, Community and Stakeholder Engagement, and Policy and Systems-Level Change. Three case exemplars that contextualize findings from the content analysis are presented.

**Conclusion:**

This work validates and extends the processes originally developed by the creators of the TSBM and offers a process-oriented example of its successful application at an external institution & CTSA hub. Co-creating TSBM Impact Profiles and documenting their development ensured that information was synthesized for broad dissemination and accessibility. Results highlight an effective process for capturing a multitude of impacts and benefits across diverse research projects with future efforts aimed at expanding the application of this method.

## Introduction

There is increased attention to making the research to practice pipeline more efficient. Prior work indicated that it takes about 17 years to turn research into practice and only about 14% of this research has been translated into real world practice (1). A more recent publication by Kahn and colleagues found similar delays in the translation of cancer prevention interventions (2). Clinical and Translational science aims to streamline this process, facilitating the uptake of research evidence in settings and communities accessed by those for whom the research is intended. It is essential to demonstrate the relevance and impact of translational research across diverse settings and contexts to successfully disseminate, implement, and sustain these evidence-based practices to positively impact communities and society as a whole (3–5). By doing so, individuals and groups within these contexts are able to gain deeper insights into the proximal and distal benefits derived from the research supporting these practices.

The Translational Science Benefits Model (TSBM) is a framework and associated tools designed to enhance the efficiency of translational science by assessing the broader clinical and community health impacts of research outcomes beyond traditional measures (4). The TSBM systematically contextualizes impact within four key domains: Clinical, Community, Economic, and Policy, identifying 30 specific health and societal benefits within these areas (4). This approach facilitates a comprehensive evaluation of clinical and translational research, enabling scientific discoveries to be translated in ways that are meaningful and relevant to audiences beyond the scientific community.

Since its inception, the TSBM has been used across a number of different research projects and public health areas to showcase potential and demonstrated benefits from these bodies of work (5–10). One notable example of this integration is the increased use of the TSBM across Clinical and Translational Science Awards (CTSA) Programs (5, 11–14) CTSA programs were established under the National Institutes of Health (NIH) National Center for Advancing Translational Science (NCATS) to support the advancement of clinical and translational science by transforming the academic research enterprise to better facilitate the translation of research discoveries into practical applications among patient and community healthcare settings (11, 15). The TSBM serves as a valuable tool to help CTSA programs evaluate the impact of clinical and translational research, support the dissemination and implementation of evidence-based interventions into practice, and guide decision-making by highlighting areas of impact (5).

While interest in the Translational Science Benefits Model (TSBM) continues to grow across the CTSA portfolio, the best approaches to effectively integrate the TSBM into clinical and translational research settings remain less well established. Identifying optimal strategies for incorporating the TSBM is essential to fully leverage its potential in capturing and communicating the real-world impacts of clinical and translational research. Further exploration and evaluation are needed to determine how the TSBM can be applied to different project stages, research goals, and community engagement practices across CTSA programs.

The University of California San Diego (UCSD) Altman Clinical and Translational Research Institute's (ACTRI) CTSA program aims to facilitate the translation of research conducted at UCSD through strategic management of the UCSD research enterprise, promoting workforce development and community engagement, generating clinical and translational science resources and pilot programs, and building innovative programs that expand the reach of clinical and translational science (National Institutes of Health, Grant UL1TR001442). In 2024, the ACTRI adopted the TSBM as a framework to evaluate the impact of UCSD's clinical and translational research efforts.

This paper describes how our team applied the TSBM to evaluate the impact of 12 diverse clinical and translational research projects and used an iterative approach to co-develop and disseminate TSBM Impact Profiles within our CTSA hub. To our knowledge, this is one of the first detailed accounts of applying the TSBM framework in this way at an institution distinct from its original developers. In doing so, we demonstrate the framework's feasibility and utility across institutional contexts. We also present findings from a content analysis conducted on the 12 profiles to identify cross-cutting themes and illustrate the process through three detailed case examples. By documenting this approach, we contribute to the growing literature on the TSBM by providing practical guidance for other institutions and reinforcing its potential as a structured tool for evaluating and disseminating translational research impact.

## Methods

The ACTRI's CTSA program utilizes the TSBM in different ways in order to examine the health and societal impact of its research projects (16). One way this is accomplished is through the creation of TSBM Impact Profiles. The concept of the TSBM Impact Profiles was initially created by the TSBM developers as a product that could facilitate the dissemination and implementation of clinical and translational research information (17). These profiles highlight research projects and corresponding health and societal benefits. Profiles are published on the ACTRI's public-facing website and are shared among the non-academic and academic communities. It was determined by the ACTRI, that the TSBM Impact Profiles would be the ideal product for the dissemination of research information due to their ability to concisely summarize complex information and the fact that they can be easily shared with different audiences. The condensed nature of the profiles was optimal for creating individual webpages for each project and allowed creative flexibility to construct visually appealing and engaging outputs.

The ACTRI TSBM Team was formed of individuals who were involved in the profile creation process. The team made up of seven individuals from the ACTRI Dissemination and Implementation Science Center (DISC) and the ACTRI Evaluation Unit, including five PhD-level and one Masters-level research faculty and staff and one graduate research intern, all with expertise in Dissemination and Implementation Science (DIS), evaluation, and the TSBM.

**Figure 1:**
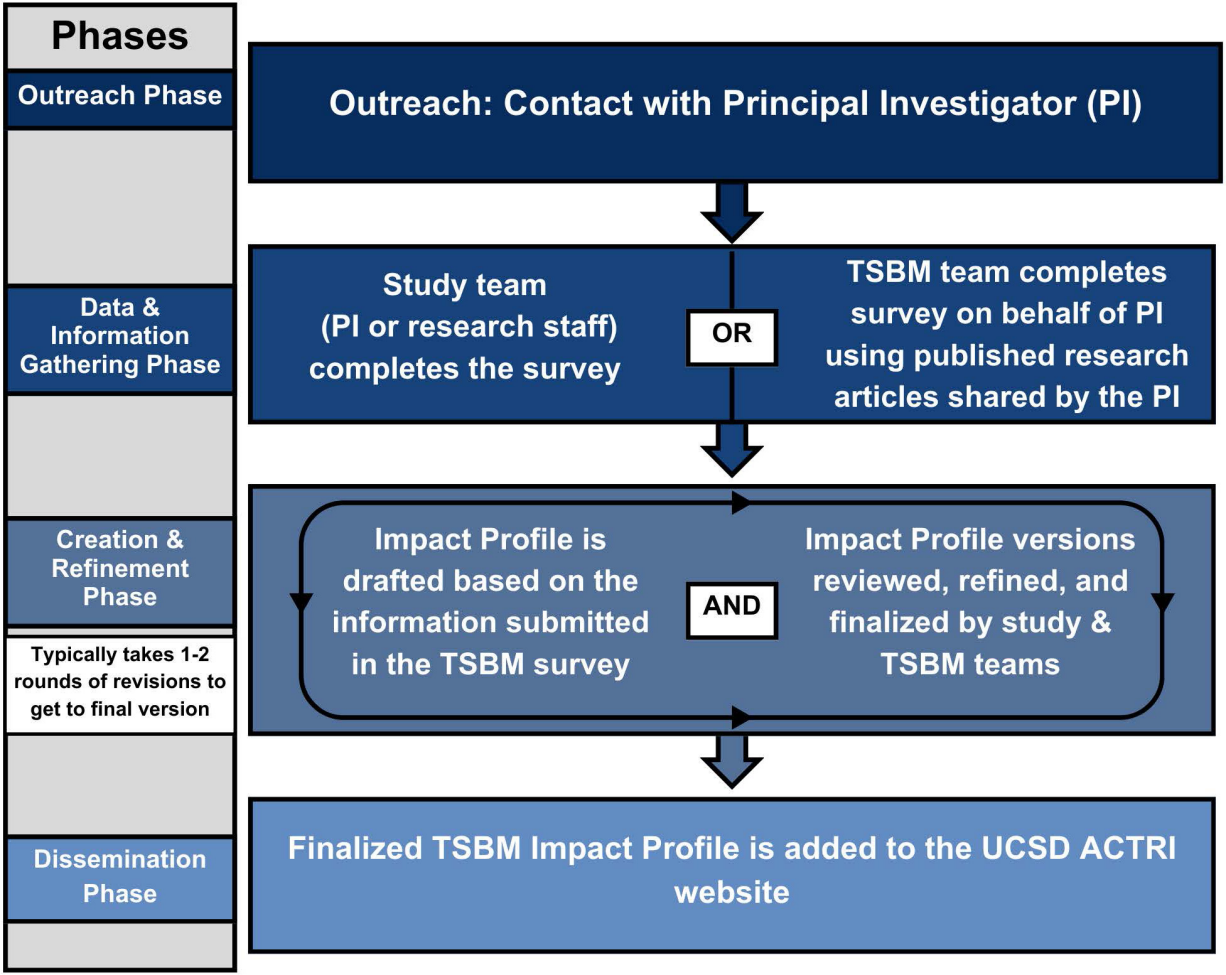
Translational science benefits model (TSBM) impact profile creation process.

The TSBM team began creating the TSBM Impact Profiles in April 2024. The process to create and disseminate the TSBM Impact Profiles spanned four phases: (1) Outreach; (2) Data & Information Gathering; (3) Creation & Refinement; and (4) Dissemination. See Figure 1 for an overview of the process.

In addition to describing the development process for the TSBM Impact Profiles, we also evaluated the content of the 12 finalized profiles. Specifically, we conducted a three-phase, Generative Artificial Intelligence (AI) (ChatGPT)-assisted content analysis to explore common themes, assess the distribution of benefits across TSBM domains, and examine differences by translational phase (18). Following the initial analysis, thematic and quantitative findings were synthesized to provide a more holistic understanding of the profiles' impacts across the TSBM domains. This evaluation aimed to illustrate the types of impacts captured through the TSBM framework and provide insight into how translational research projects manifest benefits across clinical, community, policy, and economic domains.

### Outreach

The TSBM team identified and contacted investigators to gauge interest in co-developing an impact profile for one of their research projects. Investigators were UCSD faculty associated with the ACTRI and who were the principal investigators on a research study. A convenience sampling strategy was used to identify investigators based on their direct connection to the ACTRI's KL2 program or who were affiliated with the DISC. During the outreach process, the TSBM team provided information related to the TSBM, TSBM Impact Profiles, and intended use of the profiles so that the investigator could make an informed decision on if they would like a profile created.

### Data & information gathering

An online survey was used to collect information about the research project from participating investigators (selection details described below). The survey was based on the TSBM Toolkit's Impact Profile Builder (17) and gathered information on the research project related to the challenge(s) it was addressing, the approach, intended impact, and relevant TSBM domains and benefits.

Once an investigator agreed to the creation of a TSBM Impact Profile, the survey was completed in one of two ways. The investigator (or one of their research staff) could complete the survey, or the TSBM team could complete the survey on behalf of the investigator by extracting information from various resources (e.g., research articles, protocols, websites etc.) that were provided by the investigator.

The online survey included eleven sections: (1) information about the investigator & team members (e.g., name, email, job title, role on project); (2) funding information for the project; (3) project title; (4) the challenge the project was trying to address; (5) the approach to address the challenge; (6) 2–3 high-level research highlights; (7) selection of TSBM benefits; (8) additional information about the selected benefits (e.g., indicate whether that benefit was potential or demonstrated, and provide a brief description of their rationale for choosing that benefit)); (9) the impact summary; (10) option to upload additional resources (e.g., images, publications, websites etc.); (11) consent to publish the TSBM Impact Profile on the ACTRI website. See Supplementary File 12.1 for a copy of the survey.

The survey was piloted with four investigators who provided feedback on the format and language and was refined based on the investigators' feedback. Modifications were minor and included: (1) a detailed introduction section including information about the TSBM, the importance of identifying impact and benefits from research, and the intended use of the information to support clinical and translational research and foster meaningful community engagement; (2) removal of the brief impact statement at the beginning, as it was already covered in the detailed impact section; and (3) moving the impact section to after the selection of TSBM benefits to better contextualize them. These modifications led to an informative and user-friendly interface that enabled participants, who were generally unfamiliar with the TSBM, to successfully complete the survey with minimal assistance.

In order to keep the information collected through the survey brief, word limits (maximum 200 words) were placed on the free response options (e.g., the challenge, the approach, and the impact summary). The selection of benefits section was built with display logic and organized by TSBM domain. Each benefit option contained a definition of the benefit, as defined by the TSBM developers. Once submitted, the survey answers were combined into a single PDF and emailed to the TSBM team for review. They reviewed the selected benefits and determined if they were accurately selected based on the survey respondent's rationale and the TSBM developer's definitions. If there was overlap between benefits, a clear distinction would need to be made, and consensus reached among the TSBM team in order for the benefit to be included in the TSBM Impact Profile. Uncertainty was addressed through team discussion and in rare cases reaching out to the TSBM developers for additional clarity.

### Creation and refinement

Based on the survey responses and agreed upon benefits, the TSBM team developed individual Impact Profiles. Using the TSBM developer's TSBM Impact Profile template from the Translating for Impact Toolkit as an example layout, the TSBM team populated each profile section with the relevant information and reviewed the content to ensure it was easily understood (17). This involved replacing overly technical language with terminology better suited for audiences with diverse backgrounds and education levels. ChatGPT was also used to help simplify language by inputting the technical language into the program and requesting that it produce an alternative text or supporting definition that could be read and understood at a 12th grade reading level (18). Multiple rounds of review and feedback occurred between the TSBM team and the investigator to ensure the information presented in the profiles were both accurate, comprehensive.

### Dissemination

Finalized TSBM Impact Profiles were then transformed into profile web pages on the ACTRI website (19), featuring images of the project personnel, relevant resources (i.e., websites, publications, developed tools etc.) and a downloadable PDF version of the Impact Profile.

### Evaluation of TSBM impact profiles

ChatGPT was used as a tool to facilitate initial content analysis of the Impact Profiles in order to understand common and unique themes related to the TSBM domains and benefits across projects (18, 20, 21). Prompts were co-designed by the TSBM team to optimize ChatGPT's ability to identify and collate information across TSBM Impact Profiles. Limited detail and structure in the initial prompts increased the likelihood of encountering inaccurate information (22) and response degradation in the outputs. A prompt framework (21) was adopted to guide the refinement of prompts aimed at reducing errors in outputs. Prompts were optimized by specifying the background of the task, teaching ChatGPT how to navigate the data input, ensuring the data input is consistent, and providing a detailed output template. The TSBM team purposefully did not provide guidance to ChatGPT on what themes to look for to reduce the allow for emergent patterns and reduce bias introduced by the TSBM team (21). Due to ChatGPT's conversational design, follow-up questions were used to prompt additional information and verify answers provided by ChatGPT. Once prompts were optimized, the TSBM team conducted the initial content analysis and reviewed the results for accuracy, using their in-depth knowledge of the completed TSBM Impact Profiles.

The initial content analysis was conducted in three phases, with validation checks occurring at each stage: Phase 1: Familiarization with TSBM Impact Profiles involved orienting ChatGPT to the TSBM framework and the 12 TSBM Impact Profiles. To begin, we prompted ChatGPT to summarize the TSBM, its four domains, and its 30 associated benefits using the development article by Luke et al. (4). We then uploaded a compiled PDF containing all 12 profiles and provided contextual information to help the model interpret the data structure (e.g., page numbers, section headings, and the intended purpose of the profiles). To assess the model's initial understanding, we asked ChatGPT to generate a summary of each profile, including the title, project description, identified domains, and whether the reported benefits were potential or demonstrated. We compared these outputs to the original profiles to evaluate accuracy. Where inaccuracies were identified, refinement prompts were used to correct errors and clarify domain classifications. For example, ChatGPT initially misclassified the benefit “Healthcare Delivery” under the Clinical domain rather than the Community domain, as it is currently listed in the model (4). These prompts enabled ChatGPT to revise its outputs and update its understanding accordingly. Once the summaries aligned with the original data and correct classifications, we were confident in the model's understanding of the content, which supported the decision to proceed to the content analysis phase. Prior to initiating each subsequent phase, we provided ChatGPT with the finalized results from the previous phase to ensure that accurate and validated information was used as the basis for further analysis. See Supplementary File 12.2 for ChatGPT Prompt Examples.

Phase 2: Initial Thematic Mapping with ChatGPT Assistance consisted of prompting ChatGPT to conduct a content analysis and provided instructions for it to follow. Instructions included: (1) Read each profile holistically, extracting high-level insights beyond just the TSBM domain classifications; (2) Identify and code for thematic elements; (3) For each project, note the themes present and include brief supporting evidence or examples; (4) Compare themes across profiles to identify: Themes that appear across multiple projects (common themes) and themes that are unique to a specific project or translational research phase; (5) Summarize: Common themes across profiles, themes that vary by translational phase; and provide examples from the profiles that support themes.

Following theme generation, the TSBM team conducted a manual review of ChatGPT's outputs by comparing the identified themes with the original profile content to ensure relevance, accuracy, and completeness. In cases where themes were overly broad, misaligned, or redundant, they were revised or consolidated through team discussion. For example, ChatGPT initially identified “Addressing Health Disparities” and “Healthcare Accessibility” as two separate themes, but we combined these under a broader theme of “Advancing Health Equity and Accessibility”. Similarly, a theme labeled “Community Health” was reframed as “Community and Stakeholder Engagement” to better reflect the scope of activities described across projects. This human validation step ensured that the final set of themes reflected the depth and nuance of the profile content. The list of finalized themes was shared with ChatGPT prior to Phase 3 to ensure consistency in subsequent synthesis and triangulation.

Phase 3: Synthesis and Triangulation focused on consolidating thematic and quantitative findings. ChatGPT was prompted to generate a summary table that included both qualitative themes and quantitative metrics, such as the total number of benefits, domain representation, and classification of benefits as potential or demonstrated. We also requested it to provide supporting examples and direct quotes where available. We then cross-referenced these outputs with the original profiles to validate benefit categorization and ensure thematic accuracy. For example, when ChatGPT initially reported a total of 64 benefits, rather than the correct count of 62, we prompted it to provide the number of benefits identified for each individual profile. This led to the identification of duplicated benefits in its initial output, which were subsequently corrected. While this phase did not involve formal qualitative coding, it served as a critical triangulation step that integrated both qualitative and quantitative data to ensure consistency, accuracy, and completeness.

## Results

Out of 20 investigators contacted, 12 agreed to co-create a TSBM Impact Profile for one of their research projects and eight did not respond to the outreach email, likely due to time constraints and competing demands (60% response rate). The investigators consisted of early to mid (Assistant or Associate-level) career researchers with backgrounds ranging from psychiatry and public health to nephrology and neurosciences. Four investigators were a part of the ACTRI's KL2 scholar program and eight were D&I researchers affiliated with the DISC. See Table 1 for additional information on the investigators and their research projects.

**Table 1:** Research projects and their initial characteristics included in the Altman Clinical and Translational Research Institute translational science benefits model impact profiles as of November 2024 (*n* = 12).

Project title	Principal investigator(s)	Type of research	Summary statement	Setting	Population
Access to Tailored Autism Integrated Care through Family Navigation (ATTAIN NAV)	Nicole A. Stadnick, PhD, MPH	T3 (Clinical Implementation)	ATTAIN NAV was co-designed with caregiver and healthcare partners and delivered by lay navigators to facilitate access to mental health and family support services for school-age children with autism (NIMH R34MH120190).	Pediatric Primary Care	School-age autistic children with co-occurring mental health needs.
Enhancing Collaborative Decision-Making Among Veterans of Color in VA Mental Health Care	Emily Treichler, PhD	T3 (Clinical Implementation)	This study used community-engaged mixed methods to identify the preferences, values, and current experiences related to treatment decision-making among these veterans. It also sought feedback to culturally tailor an empowerment-oriented group intervention called Collaborative Decision Skills Training, intended to boost collaborative decision-making in this group.	VA Psychosocial Rehabilitation and Recovery Center (PRRC) in Southern California	Veterans of color with serious mental illnesses in VA mental health care
Primary Prevention of Cardiovascular Disease in Patients with Elevated Lipoprotein	Harpreet Bhatia, MD, MAS, FACC	T2 (Clinical Research)	This project explores low-dose aspirin therapy as a potential method for preventing cardiovascular disease in individuals with elevated Lipoprotein(a) levels.	Multi-Ethnic Study of Atherosclerosis (MESA) Field Centers	Multi-Ethnic Study of Atherosclerosis (MESA) Field Centers
An Individualized Mental Health Intervention for Autism (AIM-HI Study)	Lauren Brookman-Frazee, PhD	T3 (Clinical Implementation)	AIM HI (An Individualized Mental Health Intervention for Autism) is a caregiver and child skill- building intervention and therapist training model for children 5 to 13 years old with autism receiving mental health services.	Publicly funded mental health services	Children with autism and caregivers
Strategies to Engage Underserved Communities in Southern California in COVID-19 Testing, Vaccinations and Trials (STOP COVID-19 Study)	Borsika Rabin, PhD, MPH, PharmD; Nicole A. Stadnick, PhD, MPH	T4 (Public Health)	The STOP COVID-19 CA UC San Diego-Global ARC project team, comprised of researchers from UC San Diego and members of the Global Action Research Center (Global ARC), aimed to identify strategies and create solutions to overcome barriers to COVID-19 testing, vaccination uptake, and participation in clinical trials, among Latino/a/x, African American, East African, Syrian, Afghan, Pacific Islanders, and South East Asian communities in San Diego County.	Federally qualified health center	Latino/a/x African American, East African, Syrian, Afghan, Pacific Islanders, and Southeast Asian communities in San Diego.
Implementation of state health insurance benefit mandates for cancer-related fertility preservation: following policy through a complex system	Irene Su, MD, MSCE; Sara McMenamin, PhD	T4 (Public Health)	This study aimed to document and understand the multi-level environment, relationships, and activities involved in using state benefit mandates to facilitate patient access to fertility preservation services.	Insurance regulators, insurers, and healthcare clinics	Young cancer patients
Community-based COVID-19 Testing Optimization for Women and Children in Underserved Areas (CO-CREATE & CO-CREATE-Ex)	Borsika Rabin, PhD, MPH, PharmD; Nicole A. Stadnick, PhD, MPH	T4 (Public Health)	CO-CREATE & CO-CREATE-Ex are linked projects aimed at providing equitable access to COVID-19 testing in medically underserved communities in central and south San Diego County through a partnership between UC San Diego, San Ysidro Health (SYH), and the Global Action Research Center (ARC).	Community health centers	Immigrant, refugee, and Black, Indigenous, People of Color (BIPOC) communities
Novel Markers for Monitoring Kidney Transplants	Clarkson Crane, MD	T2 (Clinical Research)	This project aims to improve kidney transplant outcomes by developing personalized biomarkers that predict immune responses between recipients and donors.	Transplant Center within Healthcare system	Patients receiving kidney transplants
PRISM Contextual Survey Instrument (PCSI)	James Pittman, PhD	T3 (Clinical Implementation)	The PRISM Contextual Survey Instrument (PCSI) is a 29-item survey developed to assess contextual factors that influence the implementation and sustainability of interventions in healthcare settings, helping researchers and practitioners tailor strategies to improve implementation outcomes.	Veterans Affairs Military to VA (M2VA) transition programs	Veterans
Effects of Blood Pressure on Cognition in Parkinson's Disease	Katherine Longardner, MD	T2 (Clinical Research)	This study seeks to understand how low blood pressure when standing, known as orthostatic hypotension (OH), affects cognitive performance and hemodynamics (e.g., how blood flows through your blood vessels) in people with Parkinson's disease.	Hospital/Healthcare System	Patients with orthostatic hypotension (OH)
Translating Evidence-Based Interventions for Autism (TEAMS)	Aubyn Stahmer, PhD; Lauren Brookman-Frazee, PhD	T3 (Clinical Implementation)	Developed with mental health and education partners, the TEAMS study used leadership and provider training modules to enhance autism treatment fidelity and improve child outcomes in schools and mental health settings.	Schools and mental healthcare settings	Autistic children
Mailed Colorectal Cancer Screening (ACCSIS)	Elena Martinez, PhD, MPH; Samir Gupta MD, MSCS; Scott C. Roesch, PhD	T4 (Public Health)	ACCSIS is improving colorectal cancer screening, follow-up, and referral for care among populations that have low colorectal cancer screening rates. ACCSIS focuses on underserved groups, including racial and ethnic minority populations and people living in rural or difficult-to-reach areas.	Community health centers	Low-income and minority groups

A total of 12 TSBM Impact Profiles were created using the described methods with six published on the ACTRI website and six soon to be published. Five profiles were based on investigator submitted surveys and seven surveys were based on information extracted and submitted by the TSBM team. The process used influenced the time and workflow required to complete each profile. Investigator-completed surveys allowed for greater investigator autonomy and the opportunity to draw from additional sources that may not have been readily available to the TSBM team. This process began with an initial introduction to the TSBM framework and profiles, typically requiring 1–1.5 h of communication over email or Zoom. Investigators then completed the survey independently, usually within 15–25 min. The TSBM team subsequently spent 2–3 h reviewing the submitted content, synthesizing the information into the profile template, and aligning it with the TSBM benefit domains. Follow-up communication and iterative revision cycles with the investigator added another 2 h on average. Finally, for publication on the ACTRI website the TSBM team would create a visual mock-up, send it to the investigator(s) for final review, and then build the webpage on the ACTRI's website, which required an additional 2–3 h.

In contrast, the TSBM team-led process involved the TSBM team completing the survey on behalf of the investigator based on project materials (e.g., publications, reports etc.). Initial communication, including introduction to the process and collection of relevant project documents, also required 1–1.5 h. Once materials were received, the TSBM team spent approximately 2–3 h extracting, synthesizing, and entering information into the survey instrument. This was followed by 1–2 h of follow up communication with the investigator to refine content and confirm accuracy. The final steps—creating the profile mock-up and webpage—were consistent with the investigator-led process and took 2–3 h. Across both pathways, the average total time to develop a TSBM Impact Profile was approximately 8.5 hours, with variation depending on the volume of available information, level of investigator engagement, consensus on selected benefits, and the number of content revisions required.

Based on the Translational Science Spectrum, a model that describes the process of moving research findings into clinical and community settings, the type of research varied among the TSBM Impact Profiles (14, 23). Three projects fell under T2 Research, which focuses on translating findings into patient applications by conducting controlled studies and developing evidence-based guidelines. Five projects focused on T3 Research, emphasizing translation to practice through dissemination and implementation, where research findings are applied in real-world clinical or community settings. Finally, four projects targeted T4 Research, which is concerned with community outcomes and policy impacts, aiming to influence population-level health improvements and policy reforms.

### Content analysis

A content analysis of 12 Translational Science Benefits Model (TSBM) Impact Profiles revealed a consistent set of cross-cutting themes that characterize the translational impact of diverse health research initiatives. Despite variation in focus areas, study populations, and intervention types, the following common themes emerged: Clinical Innovation and Care Integration: The majority of the TSBM Impact Profiles introduced or adapted clinical procedures, interventions, or tools for real-world care settings. These innovations spanned therapeutic procedures, diagnostic approaches, and treatment delivery models. Several profiles also contributed to the development of clinical guidelines or diagnostic tools. Advancing Health Equity and Accessibility was a central theme across all profiles. Specifically, the commitment to reducing disparities in healthcare access, especially among historically underserved populations. Profiles focused on removing multilevel barriers through strategies such as community navigation, at-home diagnostic testing, and culturally tailored outreach. These approaches enhanced care delivery to often marginalized communities. Community and Stakeholder Engagement was another key theme identified. In particular, meaningful engagement of stakeholders—patients, community members, providers, and policy actors—was noted across these profiles. Many used co-design methodologies and Community Advisory Boards, ensuring that interventions were contextually grounded, culturally relevant, and co-produced with those most impacted. Evidence-Based Implementation and Workforce Training was observed across several profiles emphasizing efforts to bridge the research-to-practice gap through training and capacity building. Profiles highlighted initiatives focused on equipping providers with skills to implement evidence-based interventions (EBIs). Policy and Systems-Level Change appeared both directly and indirectly across profiles, particularly around health policy refinement or guideline development. These included informing clinical recommendations, enhancing implementation of state mandates, and supporting advocacy training among community leaders. Lastly, Multilevel Approach to Barriers and Solutions was demonstrated across all profiles showcasing a sophisticated understanding of the complex socio-economic system affecting health outcomes. These profiles addressed challenges at the individual (e.g., patient education), organizational (e.g., clinic workflows), and systemic (e.g., reimbursement policy) levels.

### TSBM domains observed

**Figure 2:**
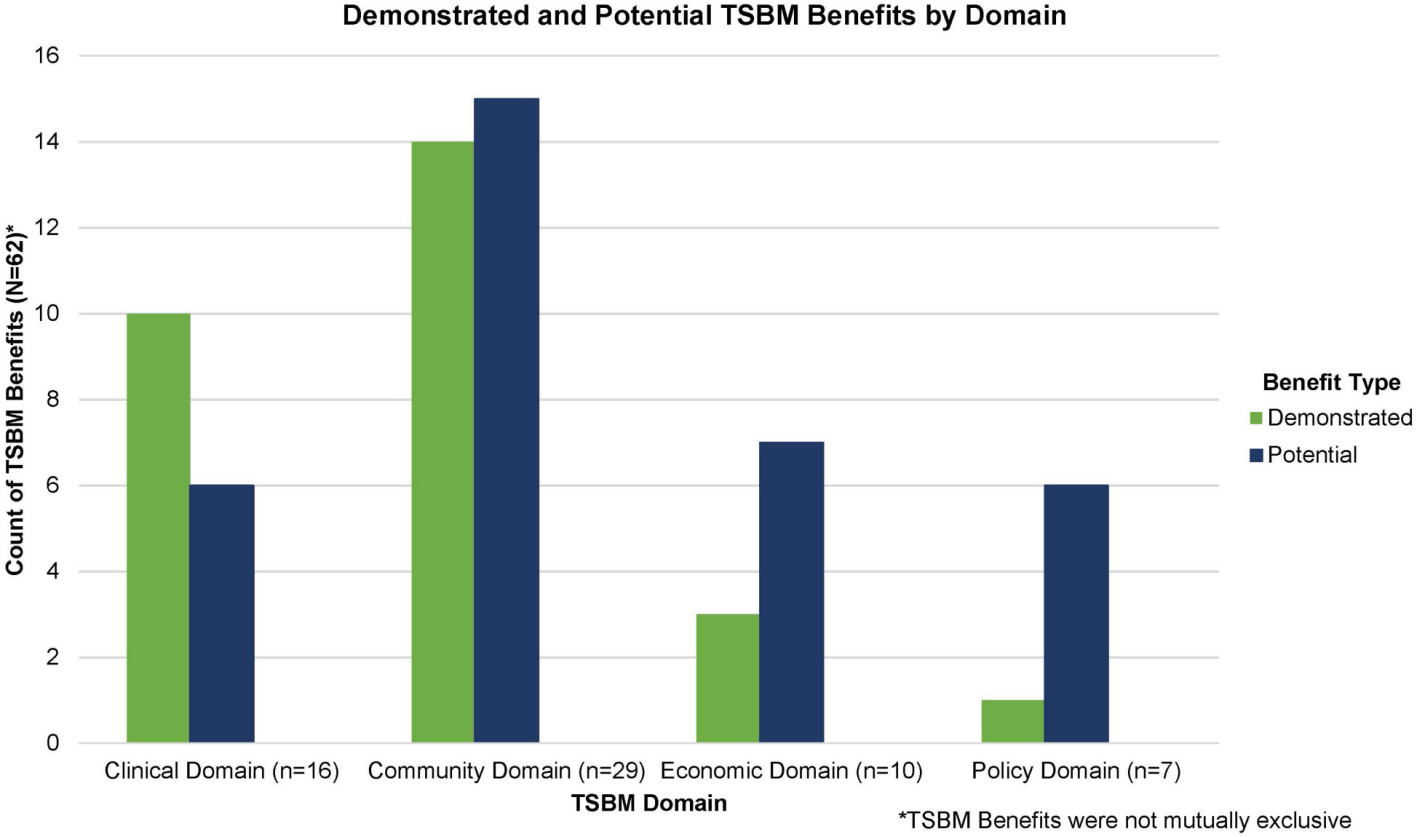
Demonstrated and potential translational science benefits across 12 sample projects by translational science benefits model (TSBM) domain.

All four TSBM domains were identified across the profiles, with the Community domain present in majority of profiles (Clinical: *n* = 10 profiles, 83.3%; Community: *n* = 12 profiles, 100%; Economic: *n* = 8 profiles, 66.7%; Policy: *n* = 7 profiles, 58.3%). A total of 62 TSBM benefits were identified across the 12 profiles, representing 19 individual benefits. On average, each profile included 5.17 benefits, with some benefits appearing in multiple profiles, as they were not mutually exclusive. The most frequently cited benefit within each domain was as follows: Therapeutic Procedures in the Clinical domain (*n* = 6 instances), Healthcare Accessibility in the Community domain (*n* = 8 instances), Cost Effectiveness in the Economic domain (*n* = 5 instances), and Policies in the Policy domain (*n* = 7 instances). Conversely, the least common benefits within each domain included Biomedical Technology in the Clinical domain (*n* = 1 instance), Healthcare Quality in the Community domain (*n* = 1 instance), Societal & Financial Cost of Illness in the Economic domain (*n* = 3 instances), and Standards in the Policy domain (*n* = 1 instance). Profiles included more potential (*n* = 34, *M* = 2.83) benefits compared to demonstrated benefits (*n* = 28, *M* = 2.33) (see Figure 2). See Table 2 for full list of benefits identified across profiles.

**Table 2:** Identified potential (P) and demonstrated (D) translational science benefits across translational science benefits impact profiles.

Project title	Clinical domain	Community domain	Economic domain	Policy domain
Access to Tailored Autism Integrated Care through Family Navigation (ATTAIN NAV)	Therapeutic Procedures (D)	Health Education Resources (D), Healthcare Accessibility (D), Community Health Services (D)		Policies (P)
Enhancing Collaborative Decision-Making Among Veterans of Color in VA Mental Health Care	Therapeutic Procedures (D), Guidelines (D), Diagnostic Procedures (D)	Healthcare Accessibility (P), Healthcare Delivery (P), Public Health Practices (P)	Cost Effectiveness (D), Societal & Financial Cost of Illness (D)	Policies (P)
Primary Prevention of Cardiovascular Disease in Patients with Elevated Lipoprotein	Therapeutic Procedures (P), Guidelines (P), Drugs (P)	Disease Prevention & Reduction (P)	Cost Savings (P)	
An Individualized Mental Health Intervention for Autism (AIM-HI Study)	Therapeutic Procedures (D)	Health Education Resources (D), Healthcare Accessibility (D), Healthcare Delivery (D), Life Expectancy & Quality of Life (D), Public Health Practices (P)	Cost Effectiveness (P)	Standards (P)
Strategies to Engage Underserved Communities in Southern California in COVID-19 Testing, Vaccinations and Trials (STOP COVID-19 Study)	Investigative Procedures (D)	Healthcare Accessibility (P), Community Health Services (P), Public Health Practices (P)	Cost Effectiveness (D), Societal & Financial Cost of Illness (P)	Policies (P)
Implementation of state health insurance benefit mandates for cancer-related fertility preservation: following policy through a complex system		Healthcare Accessibility (P), Healthcare Delivery (P)		Policies (D)
Community-based COVID-19 Testing Optimization for Women and Children in Underserved Areas (CO-CREATE & CO-CREATE-Ex)		Community Health Services (D), Healthcare Accessibility (D)	Societal & Financial Cost of Illness (P)	Policies (P)
Novel Markers for Monitoring Kidney Transplants	Biomedical Technology (D), Diagnostic Procedures (P), Guidelines (P)	Healthcare Delivery (P)	Cost Savings (P)	Policies (P)
PRISM Contextual Survey Instrument (PCSI)	Guidelines (D)	Healthcare Quality (D), Healthcare Accessibility (P)		
Effects of Blood Pressure on Cognition in Parkinson's Disease	Diagnostic Procedures (P)	Disease Prevention & Reduction (P)		
Translating Evidence-Based Interventions for Autism (TEAMS)	Therapeutic Procedures (D)	Healthcare Delivery (P), Public Health Practices (P)	Cost Effectiveness (P)	
Mailed Colorectal Cancer Screening (ACCSIS)	Diagnostic Procedures (D)	Health Education Resources (D), Healthcare Accessibility (D), Healthcare Delivery (D), Disease Prevention & Reduction (D)	Cost Savings (P)	

Three case exemplars are provided to further contextualize the content analysis findings. The first is the “Access to Tailored Autism Integrated Care through Family Navigation” (ATTAIN NAV) project, co-designed with caregiver and healthcare partners and delivered by lay navigators to facilitate access to mental health and family support services for school-age children with autism; The second is the “Strategies to Engage Underserved Communities in Southern California in COVID-19 Testing, Vaccinations and Trials” (STOP COVID-19) project aimed to identify strategies and create solutions to overcome barriers to COVID-19 testing, vaccination uptake, and participation in clinical trials, among Latino/a/x, African American, East African, Syrian, Afghan, Pacific Islanders, and South East Asian communities in San Diego County. The third is the “Enhancing Collaborative Decision-Making Among Veterans of Color in VA Mental Health Care” project aimed to enhance collaborative decision-making among Veterans of color with Serious Mental Illness (SMI) in VA mental health care.

**Figure 3:**
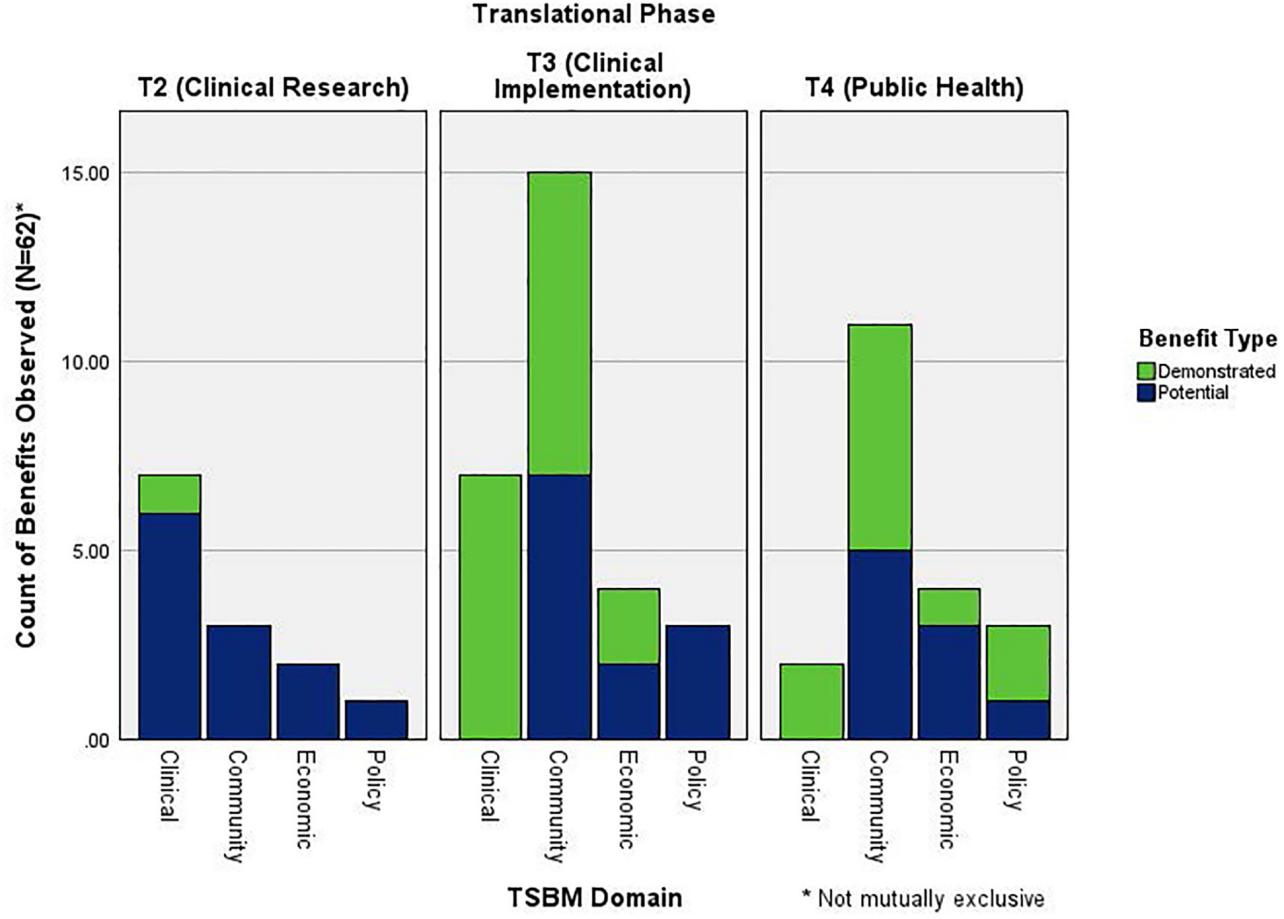
Count of TSBM benefits by translational phase and domain.

Additionally, we examined the observed TSBM benefit domains by translational research phase. The T3 (clinical implementation) phase demonstrated the greatest number of total benefits (*n* = 29), with 59% reported as demonstrated and 41% as potential. This phase showed the highest representation in the community domain (*n* = 15), including benefits such as healthcare accessibility, health education resources, healthcare delivery, and public health practices. The clinical domain was also prominent in T3 (*n* = 7), reflecting the translation of evidence-based interventions into routine practice. In contrast, the T4 (public health) phase accounted for 20 total benefits, with a slightly higher proportion of demonstrated (55%) compared to potential (45%) benefits. Like T3, the T4 phase also showed its strongest representation in the community domain (*n* = 11), reflecting a continued emphasis on community-level outcomes and interventions across later-stage translational efforts. T2 (clinical research) studies showed the fewest total benefits (*n* = 13), with only 8% demonstrated and 92% potential. These projects were primarily concentrated in the clinical domain (*n* = 7), consistent with early-phase research focused on diagnostic and therapeutic innovation. Benefits in the community (*n* = 3), economic (*n* = 2), and policy (*n* = 1) domains were limited and predominantly classified as potential (see Figure 3).

### Case examples

#### ATTAIN NAV

Observed Themes:

• Clinical Innovation and Care Integration
• Advancing Health Equity and Accessibility
• Community and Stakeholder Engagement
• Policy and Systems-Level Change

Identified Benefits:

• Clinical: Therapeutic Procedures (Demonstrated)
• Community: Health Education Resources (Demonstrated); Healthcare Accessibility (Demonstrated)
• Policy: Policies (Potential)

The ATTAIN NAV project had a significant impact on improving mental health service access for autistic children and their families through a family navigation intervention implemented in pediatric primary care (24). The project facilitated greater engagement with necessary health and community services, with 90% of families accessing at least one needed service. This clinical benefit was reflected in high levels of family satisfaction and successful connections to vital services. ATTAIN NAV empowered caregivers with the advocacy skills and knowledge necessary to navigate complex health systems more effectively. This not only facilitated access to mental health and allied health services but also enhanced families' ability to engage with care providers, helping them overcome structural barriers that often limit access to specialized care. By demonstrating the positive impact of family navigators in improving access to care, ATTAIN NAV could influence policy decisions on the role of navigation models to accelerate mental health care access and engagement. This could lead to more inclusive healthcare systems that are better equipped to serve more families. See Supplementary File 12.3 for ATTAIN NAV TSBM Impact Profile.

#### STOP COVID-19

Observed Themes:

• Advancing Health Equity and Accessibility
• Community and Stakeholder Engagement
• Policy and Systems-Level Change
• Economic and Societal Impact

Identified Benefits:

• Clinical: Investigative Procedures (Demonstrated)
• Community: Healthcare Accessibility (Demonstrated); & Public Health Practices (Demonstrated)
• Policy: Policies (Potential)
• Economic: Cost-Effectiveness (Potential) & Societal & Financial Cost of Illness (Potential)

The San Diego STOP COVID-19 CA UC San Diego-Global ARC team made significant strides in increasing awareness and understanding of factors that contribute to health disparities, focusing on improving COVID-19 testing and vaccination access for diverse groups, including Latino/a/x, African American, East African, and Asian communities (25). Their research led to better ways of reaching and helping these groups, using the findings to direct resources effectively. The team created a Theory of Change, a guide that helps others expand their services to similarly underserved populations, using methods like ethnographic documentation to understand and engage communities better. This work is crucial for providers to understand the challenges and needs of different communities, building trust and paving the way for more equitable healthcare.

The STOP COVID-19 project findings, including information gained through the Theory of Change process, have the potential to inform best practices for public health that directly relate to COVID-19 prevention, as well as other disease prevention. These actions have the potential to improve healthcare accessibility for historically underserved groups by fostering trust and increasing engagement in preventive healthcare. Additionally, the findings from STOP COVID-19 underscore the cost-effectiveness of community-based interventions and highlight the broader societal and financial cost of illness avoided through early and accessible testing services. The project's evidence-based approach has the potential to inform future health policies aimed at reducing healthcare disparities by identifying systemic barriers and tailoring solutions to meet the specific needs of immigrant and refugee communities. See Supplementary File 12.4 for STOP COVID-19 TSBM Impact Profile.

#### Enhancing collaborative decision-making among veterans of color in VA mental health care

Observed Themes:

• Clinical Innovation and Care Integration
• Community and Stakeholder Engagement
• Policy and Systems-Level Change
• Multilevel Barriers and Solutions

Identified Benefits:

• Clinical: Therapeutic Procedures (Demonstrated), Guidelines (Demonstrated), & Investigative Procedures (Demonstrated)
• Community: Healthcare Accessibility (Potential), Healthcare Delivery (Potential), & Public Health Practices (Potential)
• Policy: Policies (Potential)

The Enhancing Collaborative Decision-Making Among Veterans of Color in VA Mental Health Care project highlighted the impact of collaborative decision-making and associated patient empowerment approaches for improving mental health care for Veterans of color with serious mental illness (26). The project assessed multilevel factors including collaborative decision-making associated with satisfactory care experiences in VA mental health care. The team used community engaged strategies to work closely with Veteran partners as well as gathering mixed methods data to inform cultural tailoring of an intervention that supports Veteran-clinician collaboration. Feedback from these partners and participants also informed a new set of clinical guidelines that may improve VA mental health care accessibility and quality for Veterans of color.

The findings from this project have the potential to inform future public health practices by better incorporating the collaborative decision-making approach into mental health treatment among Veterans. By improving therapeutic procedures and public health practices that focus on Veteran engagement, the project demonstrated that involving Veterans in their care decisions can lead to more effective, tailored services. These insights have the potential to shape future healthcare policies by advocating for collaborative decision-making as a standard practice in VA mental health services. Such policy shifts would ensure that mental healthcare is more responsive and tailored to the diverse needs of Veterans, leading to greater care utilization and improved outcomes. See Supplementary File 12.5 for the Enhancing Collaborative Decision-Making Among Veterans of Color in VA Mental Health Care TSBM Impact Profile.

## Discussion

This work validates and extends the processes originally developed by the creators of the TSBM, offering a process-oriented example of its successful application at an external institution and CTSA hub. The findings highlight the TSBM's feasibility, utility, and value for broader dissemination and institutional use. By documenting the development of the TSBM Impact Profiles and presenting detailed case examples, this project offers a practical approach for operationalizing the TSBM framework to assess the health and societal impacts of clinical and translational research. These results demonstrate a replicable process for identifying and communicating a wide range of benefits across diverse research contexts. The use of concise, standardized profiles provides an accessible and engaging product for dissemination to multiple audiences. Moreover, the collaborative co-creation process with investigators ensured that complex research findings were accurately synthesized and translated into a format that is both scientifically rigorous and broadly understandable. While the TSBM Impact Profiles themselves are not new, this application demonstrates how they can be used by external institutions to support structured, co-created, and scalable dissemination of research findings. Prominent themes such as advancing health equity and access to care, supporting community-engaged research, and informing policy or systems change reflect the multidimensional nature of translational research impacts. Notably, these themes often spanned multiple TSBM domains, illustrating the interconnectedness of clinical, community, economic, and policy-level outcomes.

The consistent identification of TSBM benefits across the four domains (Clinical, Community, Economic, and Policy) underscores the model's strength in contextualizing the impacts of clinical and translational research to enhance population-wide well-being. The majority of benefits were identified within the Clinical and Community domains, suggesting that the research being conducted is applied, community-focused, and aimed at creating sustainable and equitable improvements to healthcare systems. The identification of Policy and Economic benefits, though less frequent, signals that some translational science research projects are poised to make substantial, long-term contributions by informing policy and improving economic sustainability in healthcare. This suggests that the impact of these projects may extend beyond immediate clinical or community health improvements, ultimately contributing to broader systemic change and long-term cost savings.

The distribution of benefits across translational research phases illustrates how impacts can shift as projects move from early-stage clinical research (T2) to implementation (T3) and public health application (T4). T2 studies primarily generated clinical benefits, such as diagnostic innovations and therapeutic procedures, which were largely identified as potential rather than demonstrated- highlighting the discovery-oriented nature of this phase. In contrast, T3 projects had the highest number of total benefits, with a greater proportion of demonstrated outcomes, particularly in the clinical and community domains. Reflecting a focus of T3 studies on applying and adapting evidence-based interventions in real-world service settings, where measurable impacts on access, delivery, and provider behavior are more readily observed. Similar to T3, community benefits were also predominant across T4 projects, underscoring an emphasis on improving public health through community-based strategies such as increasing access, enhancing delivery systems, and promoting public engagement. While policy and economic benefits were present, they occurred less frequently, suggesting a potential opportunity for future research to further explore and strengthen impacts within these domains.

These patterns showcase the TSBM's ability to capture phase-specific impacts and demonstrate how different types of benefits accumulate and become more visible at later stages of translation. The presence of numerous potential benefits indicates that many projects are still in development phases, collecting the necessary evidence or fine-tuning processes to achieve broader or more definitive outcomes. In contrast, demonstrated benefits signify the tangible, real-world impacts that the research projects have already achieved. This balance between potential and demonstrated benefits, suggests the importance of fostering these projects through each stage, transitioning potential benefits into demonstrated ones, to maximize their full impact.

The case exemplars highlighted the broader cross-cutting themes identified across the TSBM Impact Profiles. Advancing health equity and accessibility was central to all three case examples, with each project targeting historically underserved populations—autistic children, immigrants and refugees, and Veterans of color—using tailored, contextually grounded and community-engaged strategies. Community and stakeholder engagement were foundational, evident in co-designed interventions such as ATTAIN NAV's caregiver-partnered model, STOP COVID-19's use of a Community Advisory Board, and the Veteran-engaged design of the collaborative decision-making intervention. The projects also reflected evidence-based implementation and workforce training, especially in the integration of navigators, community educators, and culturally informed communication strategies. Additionally, each project addressed policy and systems-level change, either through identifying areas for future policy development or informing guidelines and models of care delivery. These case exemplars illustrate the multilevel, multidomain nature of translational science, capturing how community-based, clinically effective, and system-focused approaches can yield broad translational science benefits in real-world contexts.

Overall, these findings demonstrate the utility of the TSBM as a valuable tool for evaluating the impact of clinical and translational research, identifying knowledge gaps, offering a structured approach for disseminating research benefits and outcomes, and enhancing communication with stakeholders, the academic community, and the general public. This dissemination method increases the visibility and understanding of research contributions by clearly linking scientific advancements to their practical applications across clinical, community, economic, and policy settings. By showcasing tangible outcomes, this application of the TSBM serves as a dissemination framework that not only validates the importance of research investments but also fosters collaboration and support for ongoing and future initiatives.

### Limitations

The use of the TSBM to evaluate the impact of clinical and translational research, as well as the dissemination of these impacts through the TSBM Impact Profiles, shows promise as a means to improve the translation of research for various audiences. However, there are limitations to this work that we aim to address in the future. First, the primary aim of this study was to apply and evaluate the TSBM process within an institution external to the TSBM developer's institution. For this reason, the analysis was limited to profiles created within the UCSD ACTRI CTSA hub. Future work should explore cross-institutional applications of the TSBM to assess generalizability, refine dissemination strategies, and build comparative knowledge across institutions.

Second, TSBM Impact Profiles are designed to provide concise summaries, which—while accessible—may not capture all contextual details, methodological nuance, or longer-term outcomes of the projects. Moreover, the current evaluation reflects a cross-sectional snapshot of impacts. We recognize the possible value in tracking TSBM indicators longitudinally to observe how potential benefits evolve into demonstrated ones over time and aim to evaluate this with future efforts.

Third, the type of research plays a critical role in how easily information can be translated in a way that is relevant and meaningful to the public. Research situated at the earlier stages of the translational science continuum (T0, Basic Research, and T1, Preclinical) is inherently more difficult to translate into clinical or community settings, as it is further from real-world application. Our goal is to apply TSBM across all T-phases as we see opportunities to discuss translational potential throughout the continuum.

Fourth, it is crucial to consider contextual and cultural differences across target populations, particularly regarding language. For example, use of “disease” and “symptom” terminology may be inconsistent with current community preferences (e.g., in the case of autism where person-first language is prioritized). We encourage users of TSBM to adapt language to be accessible and respectful to their community audiences. In our work to date, we addressed this issue by relying on the research team's expertise in best language to describe their priority communities and topics.

Fifth, many investigators had limited knowledge of the TSBM prior to completing the survey to describe their project. As a result, the individuals completing the survey may not have been as fully informed about the TSBM, potentially impacting the quality and depth of the evaluations provided prior to review by the TSBM team. To address this concern, we worked closely with each research team and provided explanation of the TSBM impacts and examples. To enhance consistency across project, we are planning to develop trainings and continue providing technical assistance for projects.

Sixth, the use of generative AI tools such as ChatGPT introduced additional limitations. Although ChatGPT was used to support both content analysis and language simplification, all outputs underwent human review and refinement. This included not only the profile summaries but also the thematic outputs, which were critically assessed and modified by the TSBM team to ensure accuracy and completeness. Similarly, while ChatGPT was used to assist in refining plain language sections of the Impact Profiles, the resulting text sometimes lacked nuance or introduced phrasing that required revision to remain faithful to the original meaning.

Nonetheless, we acknowledge that reliance on AI-generated suggestions introduces potential bias, and that further research is needed to define best practices for integrating generative AI into qualitative methods. These limitations reflect the current constraints in AI's ability to fully grasp complex, context-specific research content. Future work should continue to explore how best to incorporate AI into qualitative research and science communication, including rigorous validation procedures, prompt design refinement, and attention to bias or overgeneralization in AI-generated language.

Finally, there were limited methods to assess the usefulness of the TSBM Impact Profiles. While website metrics are available to track how often the profiles are visited or downloaded, we have not yet been able to gather data on how community members and stakeholders perceive the usefulness of these profiles. This represents a gap in understanding their true impact and value and will be the focus of our future work.

### Future directions

This project has identified several areas for growth and expansion. First, we plan to collaborate with the UCSD ACTRI Community Advisory Board to gather feedback on the TSBM Impact Profiles. This input will help refine language, format, and content for improved relevance and accessibility across diverse audiences. Second, training and education for investigators will be central to supporting TSBM adoption. Leveraging resources from the original developers, we aim to integrate the TSBM into the research lifecycle—starting at project planning—and increase awareness of translational impact frameworks. Third, we plan to apply the TSBM to research-adjacent efforts, such as workforce development, community partnership initiatives, and training programs. These applications may help further expand the model's utility beyond traditional research outcomes. Fourth, by identifying both potential and demonstrated benefits, this work lays the foundation for future longitudinal evaluation, where projects can be re-assessed over time to track evolving impact. Finally, we will develop and implement a systematic dissemination strategy, leveraging tools such as the Dissemination Planner from the Translating for Impact Toolkit and incorporating SMART (Specific, Measurable, Achievable, Relevant, Time-Bound) goals. This strategy will aim to maximize reach, engagement, and meaningful use of TSBM Impact Profiles. Together, these efforts will strengthen the TSBM's role as a dynamic tool for evaluating, communicating, and advancing the real-world impact of translational research.

## Data Availability

The original contributions presented in the study are included in the article/Supplementary Material, further inquiries can be directed to the corresponding authors.
