# Supplementary material for: An iterative approach to evaluating impact of CTSA projects using the translational science benefits model
Source: Front Health Serv. 2025 May 20;5:1535693. doi: 10.3389/frhs.2025.1535693 (PMC12129897; doi:10.3389/frhs.2025.1535693)
Supplement: Supplementary file 1 [file Datasheet1.pdf]

# Access to Tailored Autism Integrated Care through Family Navigation (ATTAIN NAV)

Translational Science Benefits Model
**IMPACT PROFILE**

ATTAIN NAV was co-designed with caregiver and healthcare partners and delivered by lay navigators to facilitate access to mental health and family support services for school-age children with autism (NIMH R34MH120190).

## The Challenge

- Autistic children have high rates of co-occurring mental health needs, yet they face multi-level barriers to accessing and engaging with needed care[1,2,3].
- Primary care is an optimal place for early mental health needs identification and referral to mental health care.
- The Access to Tailored Autism Integrated Care (ATTAIN) study co-created an integrated mental health care model for autistic children in primary care[4].
- Pilot outcomes indicated high feasibility, acceptability, and utility but more specific and tailored implementation support was needed to expedite and secure engagement with mental health services[5,6].

## The Approach

- To address the noted challenges, the ATTAIN NAV study adapted a family navigation intervention[7] for school-age autistic children with co-occurring mental health needs.
- ATTAIN NAV was delivered in weekly sessions over 4 months by trained, lay health navigators, focused on collaborative goal development, motivational interviewing, and problem-solving strategies to promote engagement with health and community services.
- The impact of ATTAIN NAV was tested in a stepped-wedge, hybrid implementation-effectiveness study.

## The Impact

- A total of 21 primary care providers enrolled; 157 families were referred via provider or self-referral.
- A total of 65 families enrolled and 56 families completed ATTAIN NAV.
- Most families were connected to needed or requested services and caregivers reported high levels of satisfaction and benefit from ATTAIN NAV.

## RESEARCH HIGHLIGHTS

- On average, navigators completed **27 contacts** with families and spent an average of **22 minutes** per contact.
- **90% of families were successfully engaged** with at least 1 needed health or community service by navigation end.
- Families reported **high satisfaction with navigation** ($M$ = 6.31/7, $SD$ = 0.89) as well as **high satisfaction with referred services** ($M$ = 5.60/7, $SD$ = 1.32) .

## Key Benefits

CLINICAL

**Therapeutic Procedures** - *Demonstrated*
Provided an efficient and effective intervention to secure engagement with needed health and community services for autistic children.

COMMUNITY

**Health Education Resources** - *Demonstrated*
Strengthened caregiver advocacy skills and service navigation knowledge.

COMMUNITY

**Healthcare Accessibility** - *Demonstrated*
Family navigation showed potential to help address disparities in access to mental health and community-based care.

POLICY

**Policies** - *Potential*
Findings can be used as a blueprint for healthcare policies considering non-traditional mental health providers as essential members of the care teams.

---

The team: Nicole A. Stadnick[1], Kassandra Martinez-Ruezga[1], Felice Navarro[1], Pollyanna Gomez-Patino[1], Kimberly Holmquist[2], Sonya Negriff[2], Sara Walpole[2], Lauren Brookman-Frazee[1], Sarabeth Broder-Fingert[3], Miya Barnett[4], Scott Roesch[5], Joelle Robinett[1], Alondra Varela[1], & Isaac Bouchard[6]

1 University of California San Diego,
2 Kaiser Permanente, Southern California Department of Research and Evaluation
3University of Massachusetts Chan Medical School
4 University of California Santa Barbara
5 San Diego State University
6 California State University, Northridge

Contact:
Nicole Stadnick, PhD, MPH,
Department of Psychiatry UC San Diego
ATTAIN@health.ucsd.edu

Find out more:
Home | Stadnick Lab
TSBM at ACTRI

UC San Diego ALTMAN CLINICAL AND TRANSLATIONAL RESEARCH INSTITUTE
UC San Diego ACTRI Dissemination and Implementation Science Center
