# Supplementary material for: An iterative approach to evaluating impact of CTSA projects using the translational science benefits model
Source: Front Health Serv. 2025 May 20;5:1535693. doi: 10.3389/frhs.2025.1535693 (PMC12129897; doi:10.3389/frhs.2025.1535693)
Supplement: Supplementary file 2 [file Datasheet2.pdf]

Enhancing Collaborative Decision-Making Among Veterans of Color in VA Mental Health Care

Translational Science Benefits Model

IMPACT PROFILE

This study used community-engaged mixed methods to identify the preferences, values, and current experiences related to treatment decision-making among these veterans. It also sought feedback to culturally tailor an empowerment-oriented group intervention called Collaborative Decision Skills Training, intended to boost collaborative decision-making in this group.

The Challenge

Veterans of color with serious mental illness (SMI) are significantly underrepresented in decision-making processes about their treatment despite their high need for mental health services. Only 20% or less of veterans with SMI are currently involved in their treatment decisions, with even lower involvement among people of color. This project addressed the disparity in recovery-oriented services, which are crucial for supporting veterans in pursuing fulfilling lives irrespective of symptom severity

The Approach

The team engaged directly with Veterans of color, includingco-designing major study elements, such as survey selection and interview design. The research involved conducting surveys and qualitative interviews with participants. The first interview gathered insights on their experiences and preferences in treatment decision-making, while the second focused on their feedback on the Collaborative Decision Skills Training after reviewing the materials for over a week. This hands-on approach facilitated a deeper understanding of the unique needs and preferences of this group.

RESEARCH HIGHLIGHTS

- 33 Veterans were enrolled from 14 US States
- Feedback from participants suggests that Veterans see collaborative decision-making as one important component of an overall care approach that prioritizes their humanity and dignity.
- Different factors can inform satisfaction in treatment decision-making, suggesting that Veterans perceive care access and equity as fundamental precursors to ability to engage in patient-clinician collaboration.
- Veterans do not prefer that the studied intervention be tailored for Veterans of color alone or to be offered affinity groups (i.e., CDST groups for only Black Veterans). However, Veterans focused on therapist training and preparedness to provide groups in general and CDST specifically
    - For example, to be able to effectively discuss experiences of racism in mental health care; develop trust and rapport; and manage group dynamics.

The Impact

The clinical and medical benefits of this initiative focus on enhancing collaborative decision-making among Veterans of color with Serious Mental Illness (SMI) by tailoring the Collaborative Decision Skills Training based on their feedback and creating clinical guidelines from Veteran-led recommendations. This aims to improve equity in recovery-oriented mental health services within the Veterans Affairs (VA) system, and although primarily targeting mental health services, it could influence other VA services like primary care. Additionally, the community and public health benefits revolve around involving Veterans in research and public health strategies to ensure effective and equitable co-creation. Insights gained from Veteran participants help improve engagement and advocacy beyond individual decision-making, fostering stronger relationships between Veterans and VA or academic entities, ultimately enhancing public health outcomes.

Contact:

Emily Treichler, PhD
Assistant Professor
UCSD Department of Psychiatry
Principal Investigator
etreichler@health.ucsd.edu

UC San Diego ALTMAN CLINICAL AND TRANSLATIONAL RESEARCH INSTITUTE

UC San Diego ACTRI Dissemination and Implementation Science Center

Enhancing Collaborative Decision-Making Among Veterans of Color in VA Mental Health Care

Translational Science Benefits Model
IMPACT PROFILE

Key Benefits

CLINICAL

Therapeutic Procedures – *Demonstrated*
The study led to the cultural tailoring of the Collaborative Decision Skills Training manual, making it more relevant and effective for veterans of color.

CLINICAL

Guidelines – *Demonstrated*
The research helped generate veteran-led clinical recommendations, which are poised to improve the quality of care within the VA system.

CLINICAL

Investigative Procedures - *Demonstrated*
This research led to the identification of effective co-creation methodologies with Veteran partner teams.

COMMUNITY

Healthcare Accessibility - *Potential*
By identifying multilevel determinants of high-quality care, the project is set to improve the accessibility and equity of mental health services for veterans of color.

COMMUNITY

Healthcare Delivery - *Potential*
The project supports increased collaborative decision-making during care delivery by identifying key determinants and engaging the veterans in the decision-making process.

COMMUNITY

Public Health Practices- *Potential*
The identification of current levels of collaborative decision-making and its relationship with key social determinants of health among veterans of color could enhance public health practices.

Policy

Policies – *Potential*
The identification of an effective co-creation methodologies and multi-level determinants of high-quality care has the potential to inform future policies related to improved treatment of serious mental illness in veterans of color.

Additional Resources:

Treichler, E. B. H., Rabin, B. A., Cohen, A. N., & Light, G. A. (2021). How Shared Is Shared Decision Making? Reaching the Full Potential of Patient-Clinician Collaboration in Mental Health. *Harvard review of psychiatry*, *29*(5), 361–369. https://doi.org/10.1097/HRP.0000000000000304

Treichler, E. B. H., McBride, L. E., Gomez, E., Jain, J., Seaton, S., Yu, K. E., Oakes, D., Perivoliotis, D., Girard, V., Reznik, S., Salyers, M. P., Thomas, M. L., Spaulding, W. D., Granholm, E. L., Rabin, B. A., & Light, G. A. (2024). Enhancing patient-clinician collaboration during treatment decision-making: study protocol for a community-engaged, mixed method hybrid type 1 trial of collaborative decision skills training (CDST) for veterans with psychosis. *Trials*, *25*(1), 363. https://doi.org/10.1186/s13063-024-08127-4

Treichler, E. B. H., Mercado, R., Oakes, D., Perivoliotis, D., Gallegos-Rodriguez, Y., Sosa, E., Cisneros, E., Spaulding, W. D., Granholm, E., Light, G. A., & Rabin, B. (2022). Using a stakeholder-engaged, iterative, and systematic approach to adapting collaborative decision skills training for implementation in VA psychosocial rehabilitation and recovery centers. *BMC health services research*, *22*(1), 1543. https://doi.org/10.1186/s12913-022-08833-2
