# Supplementary material for: An iterative approach to evaluating impact of CTSA projects using the translational science benefits model
Source: Front Health Serv. 2025 May 20;5:1535693. doi: 10.3389/frhs.2025.1535693 (PMC12129897; doi:10.3389/frhs.2025.1535693)
Supplement: Supplementary file 3 [file Datasheet3.pdf]

Strategies to Engage Underserved Communities in Southern California in COVID-19 Testing, Vaccinations and Trials

Translational Science Benefits Model

IMPACT PROFILE

The STOP COVID-19 CA UC San Diego-Global ARC project team, comprised of researchers from UC San Diego and members of the Global Action Research Center (Global ARC), aimed to identify strategies and create solutions to overcome barriers to COVID-19 testing, vaccination uptake, and participation in clinical trials, among Latino/a/x, African American, East African, Syrian, Afghan, Pacific Islanders, and South East Asian communities in San Diego County.

The Challenge

Limited health literacy (including knowledge of the US healthcare system), lower English proficiency, and discrimination are contributing factors to poorer health outcomes, as well as barriers to accessing and receiving healthcare services, which disproportionately impact immigrant and refugee communities in the United States. The COVID-19 pandemic dramatically illustrates widening health disparities impacting immigrant, refugee, Black, Indigenous, and Communities of Color communities nationally in the United States and within specific geographic regions. These communities are significantly more likely to experience mortality and morbidity from COVID-19, along with delayed and lower testing and vaccination rates compared to white individuals in the United States.

The Approach

Representative community members from Latino/a/x, African American, East African, Syrian, Afghan, Pacific Islanders, and South East Asian communities in San Diego County were recruited to serve on a Community Advisory Board (CAB), to share their communities' concerns. Led by the Global ARC team, the CAB created a Theory of Change to inform and guide best practices and approaches for eliminating barriers to COVID-19 testing, vaccinations, and participation in research. Community members from three underserved refugee and immigrant communities in San Diego County were recruited to complete surveys that documented their needs, assets, and values, and a subset also participated in listening sessions, to elicit more detailed information regarding determinants to obtaining COVID-19 related services.

RESEARCH HIGHLIGHTS

- **The CAB identified nine necessary conditions to eliminate COVID-19 disparities** related to (1) accessible and available services; (2) culturally and linguistically competent programming; (3) investment in trusted community and faith leaders; and (4) social safety nets to provide ancillary services. The CAB defined specific actions to create these conditions and set measures to track their success.

- **Doctors/healthcare providers were rated as the most trusted messengers of COVID-19 information, however, trusted sources varied across communities.** Qualitative data from listening sessions also highlighted the importance of schools, ethnically-based community organizations, and friends and family with English literacy skills for public health information dissemination in immigrant and refugee communities.

- **A train-the-trainer policy advocacy program for ethnically-based community leaders within San Diego County was successfully developed and implemented** with participants expressing increasing levels of self efficacy for doing policy advocacy work. Engagement patterns amongst groups improved over time.

The Impact

The San Diego STOP COVID-19 CA UC San Diego-Global ARC team has made significant strides in increasing awareness and understanding of factors that contribute to health disparities, focusing on improving COVID-19 testing and vaccination access for various communities. Their research has led to better ways of reaching and helping these groups, with local collaborators and health providers using the findings to direct resources and staff effectively. The team created a Theory of Change, a guide that helps others expand their services to similarly underserved populations, using methods like ethnographic documentation to understand and engage communities better. This work is crucial for providers to understand the challenges and needs of different communities, building trust and paving the way for more equitable healthcare. The San Diego team's efforts have especially illuminated the experiences of diverse groups. By focusing on these previously less understood groups, the project has contributed to broader health equity goals, ensuring that all communities have a voice and are prioritized in health initiatives.

UC San Diego ALTMAN CLINICAL AND TRANSLATIONAL RESEARCH INSTITUTE
UC San Diego ACTRI Dissemination and Implementation Science Center

Strategies to Engage Underserved Communities in Southern California in COVID-19 Testing, Vaccinations and Trials

Translational Science Benefits Model
IMPACT PROFILE

Key Benefits

CLINICAL

Investigative Procedures - *Demonstrated*

Ethnographic approaches used in the STOP COVID-19 CA UC San Diego-Global ARC project have practical research and investigational applications, especially for populations whose needs, barriers to care and other social determinants of health are not well understood.

COMMUNITY

Public Health Practices - *Potential*

The STOP COVID-19 CA UC San Diego-Global ARC project findings, including information gained through the Theory of Change process, have the potential to inform best practices for preventive health measures directly related to COVID-19 prevention, as well as other disease prevention.

COMMUNITY

Healthcare Accessibility - *Potential*

The STOP COVID-19 CA UC San Diego-Global ARC project findings from the Theory of Change, surveys, and listening sessions inform health care providers about barriers and facilitators to access to care for immigrant and refugee communities which, if applied, can lead to improved health care practices and strategies to reach these and other underserved communities.

ECONOMIC

Cost-Effectiveness - *Demonstrated*

Information gained from the STOP COVID-19 CA UC San Diego-Global ARC project helps health service providers determine cost-effective approaches to health promotion and disease prevention efforts. For example, from the surveys and listening sessions, information was gained regarding the type of messenger who is most trusted with health advice. Utilizing this information, providers can rely on trusted spokespersons, and not waste funds for less trusted sources.

ECONOMIC

Societal & Financial Cost of Illness - *Potential*

It is anticipated that information gained from the STOP COVID-19 CA UC San Diego-Global ARC project will improve the quality of life of local immigrant, refugee, and other underserved populations, reducing the financial and social cost of disease by contributing to the delivery of more informed, thus more effective disease prevention efforts.

POLICY

Policies - *Potential*

The STOP COVID-19 CA UC San Diego-Global ARC team led the development and delivery of a five-session training for ethnically-based, immigrant and refugee community leaders to advocate for policy-level changes. Findings will inform efforts to help others increase capacity to impact policy changes and methods utilized will also inform the efforts of others with similar goals.

UC San Diego Team
Borsika Rabin, Ph.D., M.P.H., Pharm.D.: MPI; Nicole Stadnick, PhD, MPH: MPI; Kelli Cain, MA: Program Manager; Carrie Geremia, MEd: Program Coordinator; Shelia Broyles, PhD, MPH: Co-Investigator; Angel Lomeli: MPH candidate, intern

Global Action Research Center Team
Paul Watson, MS: Community PI; William “Bill” Oswald, PhD: Community PI; Marina Ibarra: Program Manager

Additional Resources

Casillas, A., Rosas, L. G., Carson, S. L., Orechwa, A., North, G., AuYoung, M., Kim, G., Guereca, J. A., Ramers, C. B., Burke, N. J., Corchado, C. G., Aguilar-Gaxiola, S., Cheney, A., Rabin, B. A., Stadnick, N. A., Oswald, W., Cabrera, A., Sorkin, D. H., Zaldivar, F., Wong, W., … Brown, A. F. (2022). *STOP COVID-19 CA:* Community engagement to address the disparate impacts of the COVID-19 pandemic in California. *Frontiers in health services*, *2*, 935297. https://doi.org/10.3389/frhs.2022.935297

Stadnick, N. A., Cain, K. L., Oswald, W., Watson, P., Ibarra, M., Lagoc, R., Ayers, L. O., Salgin, L., Broyles, S. L., Laurent, L. C., Pezzoli, K., & Rabin, B. (2022). Co-creating a Theory of Change to advance COVID-19 testing and vaccine uptake in underserved communities. *Health services research*, *57 Suppl 1*(Suppl 1), 149–157. https://doi.org/10.1111/1475-6773.13910

Stadnick, N. A., Cain, K. L., Watson, P., Oswald, W., Ibarra, M., Lagoc, R., Pezzoli, K., Laurent, L. C., Tukey, R., & Rabin, A. B. (2022). Engaging Underserved Communities in COVID-19 Health Equity Implementation Research: An Analysis of Community Engagement Resource Needs and Costs. Frontiers in health services, 2, 850427. https://doi.org/10.3389/frhs.2022.850427

Rabin, B. A., Cain, K. L., Salgin, L., Watson, P. L., Jr, Oswald, W., Kaiser, B. N., Ayers, L., Yi, C., Alegre, A., Ni, J., Reyes, A., Yu, K. E., Broyles, S. L., Tukey, R., Laurent, L. C., & Stadnick, N. A. (2023). Using ethnographic approaches to document, evaluate, and facilitate virtual community-engaged implementation research. *BMC public health*, *23*(1), 409. https://doi.org/10.1186/s12889-023-15299-2

Stadnick, N. A., Cain, K., Oswald, W., Watson, P., Nodora, J., Broyles, S., Lomeli, A., Escoto, A., Ibarra, M., Lagoc, R., & Rabin, B. (2023). Insights from Immigrant and Refugee Communities Regarding COVID-19 Needs and Opportunities: A Mixed Methods Study. *AJPM focus*, *2*(3), 100099. Advance online publication. https://doi.org/10.1016/j.focus.2023.100099

Lomeli, A., Stadnick, N., Cain, K., Watson, P., Oswald, W., Broyles, S., Marina, I., Rabin, B. (2024) "Increasing capacity for ethnically-based community leaders to engage in policy change: Assessing the impact of using the Train the Trainer approach" BMC Public Health, in press.

NIH Community Engagement Alliance (CEAL) - https://ceal.nih.gov/

The Global Action Research Center (Global ARC) - https://www.theglobalactionresearchcenter.org/

STOP COVID-19 Informational Video - https://youtu.be/BU1wZ1NrvNI?si=70nn1j5ga4PF98lh

STOP COVID-19 Informational Video (Short Version) - https://youtu.be/pVgOKDxfvIg?si=4C_kySxbl8h0DWz2

Find out more:
https://actri.ucsd.edu/centers-services/portfolio/disc/stop-covid19/index.html#Resources-and-Tools

Contact:
Kelli Cain (Project Manager): kcain@health.ucsd.edu
