# Supplementary material for: An iterative approach to evaluating impact of CTSA projects using the translational science benefits model
Source: Front Health Serv. 2025 May 20;5:1535693. doi: 10.3389/frhs.2025.1535693 (PMC12129897; doi:10.3389/frhs.2025.1535693)
Supplement: Supplementary file 4 [file Datasheet4.pdf]

UC San Diego
ACTRI Dissemination and
Implementation Science Center

Translational Science Benefits Model: Impact Profile

This form was adapted from the original TSBM Impact Profile Builder, developed by the TSBM developers at Washington University in St. Louis.

This form will help you create a succinct summary (or Impact Profile) of the impact of your research. The information submitted in this form will be used by the TSBM team to create your Impact Profile.
Click here to view our current Impact Profiles published on the ACTRI website!
You can use your Impact Profile to demonstrate your impact to the community or general public, demonstrate your impact for promotion and tenure decisions, summarize your research progress to study participants or supervisors, highlight the value of your work for policymakers, or share with media contacts for news stories.In order to demonstrate the health and societal benefits of clinical and translational research, the Translational Science Benefits Model (TSBM) was developed as a tool for researchers, administrators, and policymakers to measure the impact of their work in four distinct domains: **CLINICAL**, **COMMUNITY**, **ECONOMIC**, and **POLICY**. The TSBM showcases 30 distinct and tangible benefits stemming from translational research. **Clinical benefits** include new biomedical technologies and therapeutic procedures **Community benefits** include health education resources and improved access to health care **Economic benefits** include increased cost effectiveness and new commercial entities **Policy benefits** include legislation and organizational policies.

[Embedded Informational Video: https://youtu.be/7C9ccZIa7kA]

Contact Information

Name

First Name　　Last Name

Email

example@example.com

Primary Institutional Affiliation/ACTRI Unit

If applicable, additional team members, roles, & affiliations (e.g. community partners, mentors who supported your project, co-investigators, research assistants etc.)

Please cite any funding that help contribute & fund this project:

Plain Language Project Title

Write a very short (3-5 word) title for your project that you can use to quickly communicate about your work. Use active verbs to describe change.

Project Title

Summary Statement

Write one sentence to help the average person understand what your project is about.

Summary Statement

Example: ATTAIN NAV was co-designed with caregiver and healthcare partners and delivered by lay navigators to facilitate access to mental health and family support services for school-age children with autism

The Challenge

In 1-3 sentences, describe the issue or problem that your research addresses. Use simple terms that people without technical knowledge can understand.

The Challenge

The Approach

List what you are doing or plan to do to address the problem. Focus on what makes your project unique and innovative. Identify 3-4 three key activities and list in bullets.

The Approach

0/200

Research Highlights

If applicable: Use this space to highlight 2-3 of the most important impacts of your project. Use quantitative impacts, when possible, for example: x people served, $x cost savings, x% improvement on a key outcome. For certain projects, research highlights may include descriptive statements with highlighted key words instead of numbers.

Research Highlights

Key Benefits

For your Impact Profile, select the most important 3-5 benefits of your project to date. You can select any benefits across any of the 4 domains (Clinical, Community, Economic, and Policy).
Once you have selected your project's benefits, you will be prompted to tell us a bit more about them!

Clinical

Clinical and Medical Benefits

(Procedures & guidelines, Tools & products)

Clinical and Medical Benefits

- ☐ Diagnostic Procedures- Methods and techniques performed to diagnose disease, disorders, or conditions
- ☐ Investigative Procedures- Research methods used in preclinical, clinical, and other scientific studies
- ☐ Clinical Guidelines- Formal recommendations or principles to assist with patient care for specific clinical circumstances
- ☐ Therapeutic Procedures- Methods and techniques that pertain to interventions, treatment, or prevention of diseases, disorders, or conditions
- ☐ Biological Factors and Products- Biological substances used to indicate, diagnose, prevent, or treat diseases or medical conditions
- ☐ Biomedical technologies- Technology applications for measuring, diagnosing, and treating health conditions, including tools, methods, strategies, and devices
- ☐ Drugs- Pharmaceutical products for human or veterinary use intended to diagnose, treat, cure, or prevent health conditions
- ☐ Clinical Equipment or Supplies- Apparatus, instruments, and materials for diagnostic, surgical, therapeutic, and scientific procedures
- ☐ Software Technologies- Computer programs or software installed on mobile or other electronic devices

Community

Community and Public Health Benefits

(Health activities & products, Health care characteristics, Health promotion)

Community and Public Health Benefits

- ☐ Community Health Services- Diagnostic, therapeutic, and preventive health services provided for individuals in a community
- ☐ Consumer Software- Digital and mobile technologies used by or for consumers to improve health care delivery and outcomes
- ☐ Health Education Resources- Educational resources that lead to the improvement of health of individuals, populations, or communities
- ☐ Health Care Accessibility- Increased equity and ability for all to gain entry to and to receive services from the health care system, regardless of race, ethnicity, age, income, ability, sex, gender, sexual orientation, geographic location, or health status
- ☐ Health Care Delivery- Improved provision and distribution of health services to a patient population
- Health Care Quality- Improved general characteristics and quality of the health service or care provided

☐

based on accepted standards of quality

☐ Disease Prevention & Reduction- Resources that enhance health promotion and disease prevention in communities or populations

☐ Life Expectancy & Quality of Life- Improvement in the average age of death or how often illness and injury impedes everyday life for a particular population

☐ Public Health Practices- Organization or delivery of public health services benefits to communities or populations

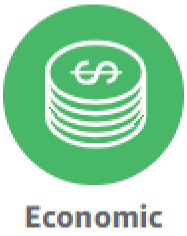

Economic Benefits

(Commercial products, Financial savings & benefits)

Economic Benefits

☐ License Agreements- Governmental permits based on intellectual property

☐ Non-Profit or Commercial Entities- Creation of businesses or non-profit organizations

☐ Patents- Government authority or licenses based on intellectual property

☐ Cost-Effectiveness- Improvement in the benefits of a program relative to its cost

☐ Cost Savings- Reduced financial costs of services or goods to providers or consumers

☐ Societal and Financial Cost of Illness- Reduced social and economic costs of acute or chronic disease or other health conditions

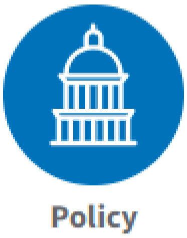

Policy and Legislative Benefits

(Advisory activities, Policies & legislation)

Policy and Legislative Benefits

☐ Committee Participation- Participation in advisory, standards, or other governmental or nongovernmental committees

☐ Expert Testimony- Formal presentation of data or results to governmental, judicial or other regulatory bodies

☐ Scientific Research Reports- Non-technical, evidence-based documents geared toward audiences who intend to use the information for policy/behavioral change

☐ Legislation- Bills, laws, statutes, and ordinances passed through formal legislative bodies such as congress, parliaments, state or provincial legislatures, and county and city councils

☐ Policies- Procedural rules formally adopted and mandated by governmental agencies or private or non-profit organizations

☐ Standards- Formal designations of levels of quality defined by industry, occupational groups, or governmental bodies

Tell us about the benefits you selected

<u>**Are these Potential benefits or Demonstrated benefits?**</u>

Potential Benefits: These are anticipated or projected impacts that a research project aims to achieve as it develops. They represent goals that have been identified but are not yet fully realized. Potential benefits indicate ongoing work, such as early-stage interventions, pilot studies, or research that is still gathering evidence to support its broader impact.

Demonstrated Benefits: These are tangible, real-world outcomes that have already been achieved as a result of the research. Demonstrated benefits provide evidence of effectiveness in specific settings, populations, or policies. These impacts have been measured, observed, or successfully implemented, showcasing how the research has translated into meaningful changes in clinical, community, economic, or policy domains.

<u>**Example of how we use this data for your Impact Profile:**</u>

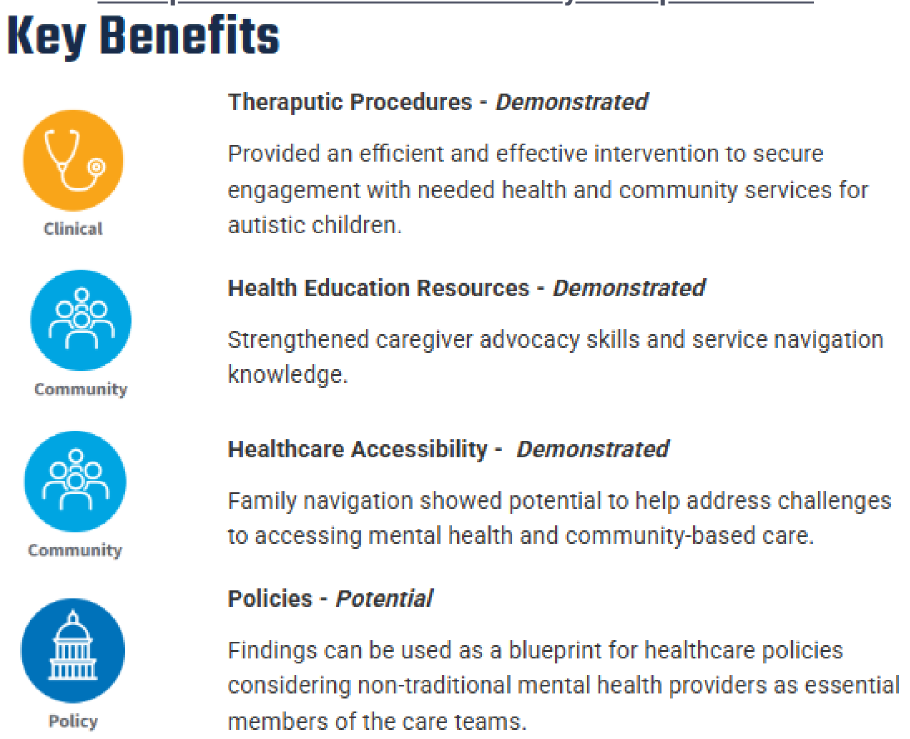

Note: The questions below are programmed with display logic and only appear if the specific benefit was selected above.

Please indicate whether the benefit associated with Diagnostic Procedures is a potential benefit or the benefit has been demonstrated through this project.

- ◯ Potential
- ◯ Demonstrated

Please describe why you selected the diagnostic procedures benefit.

Please indicate whether the benefit associated with Investigative Procedures is a potential benefit or the benefit has been demonstrated through this project.

- Potential
- Demonstrated

Please describe why you selected the investigative procedures benefit.

Please indicate whether the benefit associated with Clinical Guidelines is a potential benefit or the benefit has been demonstrated through this project.

- Potential
- Demonstrated

Please describe why you selected the clinical guidelines benefit.

Please indicate whether the benefit associated with Therapeutic Procedures is a potential benefit or the benefit has been demonstrated through this project.

- Potential
- Demonstrated

Please describe why you selected the therapeutic procedures benefit.

Please indicate whether the benefit associated with Biological Factors and Products is a potential benefit or the benefit has been demonstrated through this project.

- ◯ Potential
- ◯ Demonstrated

Please describe why you selected the biological factors and products benefit.

Please indicate whether the benefit associated with Biomedical Technology is a potential benefit or the benefit has been demonstrated through this project.

- ◯ Potential
- ◯ Demonstrated

Please describe why you selected the biomedical technology benefit.

Please select the option that best fits with the following key benefit: Drugs

- ◯ Potential
- ◯ Demonstrated

Please describe why you selected the drugs benefit.

Please indicate whether the benefit associated with Equipment & Supplies is a potential benefit or the benefit has been demonstrated through this project.

- ◯ Potential
- ◯ Demonstrated

Please describe why you selected the equipment and supplies benefit.

Please indicate whether the benefit associated with Software Technologies is a potential benefit or the benefit has been demonstrated through this project.

- ◯ Potential
- ◯ Demonstrated

Please describe why you selected the software technologies benefit.

Please indicate whether the benefit associated with Community Health Services is a potential benefit or the benefit has been demonstrated through this project.

- ◯ Potential
- ◯ Demonstrated

Please describe why you selected the community health services benefit.

Please indicate whether the benefit associated with Consumer Software is a potential benefit or the benefit has been demonstrated through this project.

- Potential
- Demonstrated

Please describe why you selected the consumer software benefit.

Please indicate whether the benefit associated with Health Care Accessibility is a potential benefit or the benefit has been demonstrated through this project.

- Potential
- Demonstrated

Please describe why you selected the Health Care Accessibility benefit.

Please indicate whether the benefit associated with Health Education Resources is a potential benefit or the benefit has been demonstrated through this project.

- Potential
- Demonstrated

Please describe why you selected the health education resources benefit.

Please indicate whether the benefit associated with Health Care Delivery is a potential benefit or the benefit has been demonstrated through this project.

- ◯ Potential
- ◯ Demonstrated

Please describe why you selected the health care delivery benefit.

Please indicate whether the benefit associated with Healthcare Quality is a potential benefit or the benefit has been demonstrated through this project.

- ◯ Potential
- ◯ Demonstrated

Please describe why you selected the health care quality benefit.

Please indicate whether the benefit associated with Disease Prevention and Reduction is a potential benefit or the benefit has been demonstrated through this project.

- ◯ Potential
- ◯ Demonstrated

Please describe why you selected the disease prevention and reduction benefit.

Please indicate whether the benefit associated with Life Expectancy and Quality of Life is a potential benefit or the benefit has been demonstrated through this project.

- ◯ Potential
- ◯ Demonstrated

Please describe why you selected the life expectancy and quality of life benefit.

Please indicate whether the benefit associated with Public Health Practices is a potential benefit or the benefit has been demonstrated through this project.

- ◯ Potential
- ◯ Demonstrated

Please describe why you selected the public health practices benefit.

Please indicate whether the benefit associated with License Agreements is a potential benefit or the benefit has been demonstrated through this project.

- ◯ Potential
- ◯ Demonstrated

Please describe why you selected the license agreements benefit.

Please indicate whether the benefit associated with Non-Profit or Commercial Entities is a potential benefit or the benefit has been demonstrated through this project.

- ◯ Potential
- ◯ Demonstrated

Please describe why you selected the non-profit or commercial entities benefit.

Please indicate whether the benefit associated with Patents is a potential benefit or the benefit has been demonstrated through this project.

- ◯ Potential
- ◯ Demonstrated

Please describe why you selected the patents benefit.

Please indicate whether the benefit associated with Cost Effectiveness is a potential benefit or the benefit has been demonstrated through this project.

- ◯ Potential
- ◯ Demonstrated

Please describe why you selected the cost effectiveness benefit.

Please indicate whether the benefit associated with Public Health Practices is a potential benefit or the benefit has been demonstrated through this project.

- ◯ Potential
- ◯ Demonstrated

Please describe why you selected the cost savings benefit.

Please indicate whether the benefit associated with Societal and Financial Costs of Illness is a potential benefit or the benefit has been demonstrated through this project.

- ◯ Potential
- ◯ Demonstrated

Please describe why you selected the societal and financial cost of illness benefit.

Please indicate whether the benefit associated with Committee Participation is a potential benefit or the benefit has been demonstrated through this project.

- ◯ Potential
- ◯ Demonstrated

Please describe why you selected the committee participation benefit.

Please indicate whether the benefit associated with Expert Testimony is a potential benefit or the benefit has been demonstrated through this project.

- ◯ Potential
- ◯ Demonstrated

Please describe why you selected the expert testimony benefit.

Please indicate whether the benefit associated with Scientific Research Reports is a potential benefit or the benefit has been demonstrated through this project.

- ◯ Potential
- ◯ Demonstrated

Please describe why you selected the scientific research reports benefit.

Please indicate whether the benefit associated with Legislation is a potential benefit or the benefit has been demonstrated through this project.

- ◯ Potential
- ◯ Demonstrated

Please describe why you selected the legislation benefit.

Please indicate whether the benefit associated with Policies is a potential benefit or the benefit has been demonstrated through this project.

◯ Potential
◯ Demonstrated

Please describe why you selected the policies benefit.

Please indicate whether the benefit associated with Standards is a potential benefit or the benefit has been demonstrated through this project.

◯ Potential
◯ Demonstrated

Please describe why you selected the standards benefit.

Briefly summarize the impact of your project

Reflecting on both your project as a whole and the TSBM benefits you selected above, please describe the overall impact of your project. For example, how has it benefited, or how will it benefit, the field, community, and/or society as a whole? Be as clear as possible about who has benefited or will benefit in the future from your research.

Impact Summary

Consent to publish Impact Profile on ACTRI Website:

The TSBM Impact Profiles showcase the potential and demonstrated benefits of our research across community, clinical, economic, and policy sectors. These profiles are intended to be a resource that community members and researchers can use to learn more about the research being done at the ACTRI, societal and research-related benefits that stem from this research, and to foster knowledge sharing among the public and research communities.
IMPORTANT: You will have multiple opportunities to review and provide feedback on your project's Impact Profile prior to it being published to ensure the accuracy of the information. We will never publish your Impact Profile without your approval.
Click here to view our current Impact Profiles published on the ACTRI website!

Once your Impact Profile is finalized by both you and the TSBM team, do we have your permission to publish your Impact Profile on the ACTRI website?

- ◯ Yes
- ◯ No

Submit

If available, please upload any additional documents that could be used for the impact profile (e.g., publications, team images, additional resources etc.)

Browse Files

Drag and drop files here
