# Supplementary material for: An iterative approach to evaluating impact of CTSA projects using the translational science benefits model
Source: Front Health Serv. 2025 May 20;5:1535693. doi: 10.3389/frhs.2025.1535693 (PMC12129897; doi:10.3389/frhs.2025.1535693)
Supplement: Supplementary file 5 [file Datasheet5.pdf]

Content Analysis

Phase 1: Familiarization with TSBM Impact Profiles

- [Prompt 1] *This document describes the Translational Science Benefits Model and the 30 different benefits that can come from clinical and translational research. Benefits are categorized across 4 domains: Clinical, Community, Economic, and Policy. Please review this document and list each benefit with its definition that falls within each domain.* [included attachment of TSBM manuscript]
  - TSBM Manuscript used: Luke, D.A. et al. (2018) 'The Translational Science Benefits Model: A New Framework for Assessing the Health and Societal Benefits of Clinical and Translational Sciences', *Clinical and Translational Science*, 11(1), pp. 77–84. https://doi.org/10.1111/cts.12495
  - [Human validation to check that benefits were correctly listed and defined within each domain- compare to manuscript]
- [Prompt 2] *We created 12 TSBM Impact Profiles. TSBM Impact profiles are brief summaries of clinical and translational research and discusses the health and societal benefits of this research based on the TSBM. You can use Impact Profile to demonstrate your impact to the community or general public. We would like to use ChatGPT to conduct a directed content analysis of these 12 profiles. The profiles follow a similar structure with the title of the profile at the top left corner, the summary statement below, the challenge the project is addressing on the left side, the approach the project is taking to address the challenge on the left side, the impact of the project towards the bottom of the page, research highlights from the projects on the right side in the blue box, and the identified TSBM benefits listed under "Key Benefits". The specific benefits are written in bold and the domain is illustrated by the icon to the left of the benefit (Clinical, Community, Policy, and Economic).*
  - ChatGPT updates its knowledge

- [Prompt 3] *This PDF includes 12 individual TSBM Impact Profiles. Page 1 is the profile for the ATTAIN NAV study; Pages 2-3 is the profile for the Shared Decision Making Among Veterans profile; Pages 4-5 is the profile for Primary Prevention of Cardiovascular Disease in Patients with Elevated Lipoprotien; Pages 6-8 is the profile for the AIM-HI study; Pages 9-10 is the profile for the STOP COVID-19 study; Page 11-12 is the profile for Implementation of state health insurance benefit mandates; Pages 13-15 is the profile for CO-Community-based COVID-19 Testing Optimization; Pages 16-17 is the profile for Novel Markers for Monitoring Kidney Transplants; Pages 18-19 is the profile for the PRISM Contextual Survey Instrument (PCSI); Pages 20-21 is the profile for Effects of Blood Pressure on Cognition; Pages 22-23 is the profile for the Translating Evidence-Based Interventions for Autism (TEAMS); Pages 24-25 is the profile for the Mailed in Colorectal Cancer Screening study.*

The profiles follow a similar structure with the title of the profile at the top left corner, the summary statement below, the challenge the project is addressing on the left side, the approach the project is taking to address the challenge on the left side, the impact of the project towards the bottom of the page, research highlights from the projects on the right side in the blue box, and the identified TSBM benefits listed under "Key Benefits". The specific benefits are written in bold and the domain is illustrated by the icon to the left of the benefit (Clinical, Community, Policy, and Economic).

Please review the attached document and summarize the key information in each profile in a table.

1. *Code each profile by noting instances of specific TSBM benefits and whether they are potential or demonstrated organized by TSBM domains (Clinical, Community, Economic, and Policy).*
2. *Include the specific profiles where the benefit is identified & the total number of profiles each benefit is identified.*
3. *Include the number of benefits within each domain.*
4. *Include the number of potential and demonstrated benefits within each domain.*
5. *Include brief description of the profile content*
6. *Create a table summarizing the results. Only include benefits that are listed in the profiles. Do not add or assume additional benefits are included. Make sure that the correct benefits are listed in the correct domains. The table should include the following columns and information:*
   - *Project Title*
   - *Brief description of profile*
   - *Clinical Domain (please list specific TSBM benefits that are identified in the profile that fall under the clinical domain. If there are none identified in the project, leave blank. If the benefit is potential include "(P)" at the end of the benefit name. If the benefit is demonstrated include a "(D)" at the end of the benefit name.)*
   - *Community Domain (please list specific TSBM benefits that are identified in the profile that fall under the community domain. If there are none identified in the project, leave blank. If the benefit is potential include "(P)" at the end of the benefit name. If the benefit is demonstrated include a "(D)" at the end of the benefit name.)*
   - *Economic Domain (please list specific TSBM benefits that are identified in the profile that fall under the economic domain. If there are none identified in the project, leave blank. If the benefit*

is potential include "(P)" at the end of the benefit name. If the benefit is demonstrated include a "(D)" at the end of the benefit name.)

Note: Benefits are not mutually exclusive. A single profile may list multiple benefits within the same or different domains, and each instance should be counted independently. Only include benefits that are listed in the profiles. Do not add or assume additional benefits are included.

- Attached PDF of combined TSBM Impact Profiles
- [Human validation] Team compared generated table to the original profiles. Benefits were evaluated to make sure they were correctly. categorized and applied to the respective profiles (e.g., look at type of benefit, number of benefits, domain etc.)
- Finalized content was used to create Tables 1 & 2 for the manuscript.

Human Validation: Examples of refinement prompts

- [Phase 1 Refinement Prompt 1] *The benefit "Healthcare Delivery" does not fall under Clinical benefits, it is a Community benefit according to the TSBM framework.*
  - ChatGPT updates knowledge and updates table
- [Phase 1 Refinement Prompt 2] *For the TSBM Impact Profile "Enhancing Collaborative Decision-Making Among Veterans of Color in VA Mental Health Care" the benefit "Policies" is incorrectly labeled as a "Demonstrated" benefit. It should be labeled as a "Potential" benefit according to the profile.*
  - ChatGPT updates knowledge and updates table

Share final summary results with ChatGPT

(Ensures ChatGPT uses correct information for next phase)

- [Prompt 1] *Attached are the descriptive tables of the profiles based on your corrected summary. We have also included in Table 1 the translational research phase that each profile fit under.*
  - Attachments Table 1 & Table 2
  - ChatGPT updates its knowledge

Phase 2: Initial Thematic Mapping with ChatGPT Assistance

[Prompt 1] *I would like you to conduct a qualitative thematic analysis of these 12 TSBM Impact Profiles to identify and synthesize common and unique themes.*

Use the full text and structure of the TSBM Impact Profiles from the above PDF file, focusing on the following elements per profile:

- *Project summary*
- *The challenge*
- *The approach*

- *The impact*
- *Research highlights*
- *Identified TSBM benefits*
- *Key benefits by domain*

Instructions:

1. *Read each profile holistically, extracting high-level insights beyond just the TSBM domain classifications.*
2. *Identify and code for thematic elements.*
3. *For each project, note the themes present and include brief supporting evidence or examples.*
4. *Compare themes across profiles to identify:*
    a. *Themes that appear across multiple projects (common themes)*
    b. *Themes that are unique to a specific project or translational research phase (use Table 1 to identify translational research phase)*
5. *Summarize:*
    a. *Common themes across profiles*
    b. *Themes that vary by translational phase*
    c. *Examples from the profiles that support themes*

- [Human validation] Team reviews themes to make sure they are accurate.

Human Validation: Examples of refinement prompts

- [Phase 2 Refinement Prompt 1] *The themes "Addressing Health Disparities" and "Healthcare Accessibility" are presented as two separate themes. However, these can be combined into a broader theme of "Advancing Health Equity and Accessibility".*

- [Phase 2 Refinement Prompt 2] *The theme "Community Health" seems too broad and not reflective of the specific community-engagement activities reported in the profiles. "Community and Stakeholder Engagement" is a more accurate theme.*

Share final summary results with ChatGPT

(Ensures ChatGPT uses correct information for next phase)

- [Prompt 1] *These are the final themes identified.*
    - ChatGPT updates its knowledge

Phase 3 Synthesis and Triangulation

[Prompt 1] *Consolidate and synthesize the qualitative themes and quantitative metrics identified across 12 TSBM Impact Profiles to ensure consistency, completeness, and alignment between narrative content and structured benefit data.*

Instructions

1. *Review previously extracted data from the TSBM Impact Profiles, including:*
   - *Thematic analysis results (e.g., themes such as Health Equity, Community Engagement, Policy Impact)*
   - *Quantitative benefit data (e.g., total benefit counts, domains, status as Demonstrated or Potential)*
   - *Translational research phase mappings (from Table 1)*
   - *Specific benefit types per domain (from Table 2)*
2. *For each profile, generate a summary entry that includes:*
   - *Translational phase (T2, T3, or T4)*
   - *Total number of benefits identified*
   - *Breakdown by domain (Clinical, Community, Economic, Policy)*
   - *Count of Demonstrated vs. Potential benefits*
   - *Qualitative themes identified (use* final thematic coding results)
   - Include supporting examples or direct quotes from the profile (e.g., *excerpts from "impact" or "research highlights" sections)*
3. *Ensure each summary accurately reflects both the quantitative data and the narrative content. If discrepancies are noted (e.g., a theme mentioned in the write-up but not represented in the benefits table), flag for review.*

- [Human validation] Team reviews quantitative results and themes to make sure they are accurate.

Human Validation: Examples of refinement prompts

- [Phase 3 Refinement Prompt 1] *The benefit "Healthcare Quality" does not fall under Clinical benefits, it is a Community benefit according to the TSBM framework.*
  - ChatGPT updates knowledge and updates output
- [Phase 3 Refinement Prompt 2] *The theme "Policy and Systems-Level Change" is present across 7 profiles, not 10.*
  - ChatGPT updates knowledge and updates output
- [Phase 3 Refinement Prompt 3] *There should be a total of 62 identified benefits, however you listed 64. Please list number of benefits identified for each profile that resulted in 64 benefits being listed.*
  - ChatGPT updates knowledge and updates output

TSBM Impact Profile Language Refinement

[Prompt 1] *Refine the language of this TSBM Impact Profile so that it is clear, engaging, and accessible to non-academic audiences, including community members, policymakers, funders, and practitioners. The revised version should maintain accuracy while avoiding technical jargon and dense academic phrasing, while keeping the reading level at approximately the 12th-grade level.*

Instructions:

- *Simplify complex sentences and remove academic jargon*
- *Replace technical terms with plain language alternatives, or briefly define them if needed*
- *Preserve the main points and integrity of the content (e.g., what the project is, why it matters, what it achieved)*
- *Use an active voice and a clear, engaging tone*
